# Supplementary figures and images for: High-throughput phenotyping of infection by diverse microsporidia species reveals a wild C. elegans strain with opposing resistance and susceptibility traits
Source: PLoS Pathog. 2023 Mar 9;19(3):e1011225. doi: 10.1371/journal.ppat.1011225 (PMC10030041; doi:10.1371/journal.ppat.1011225)

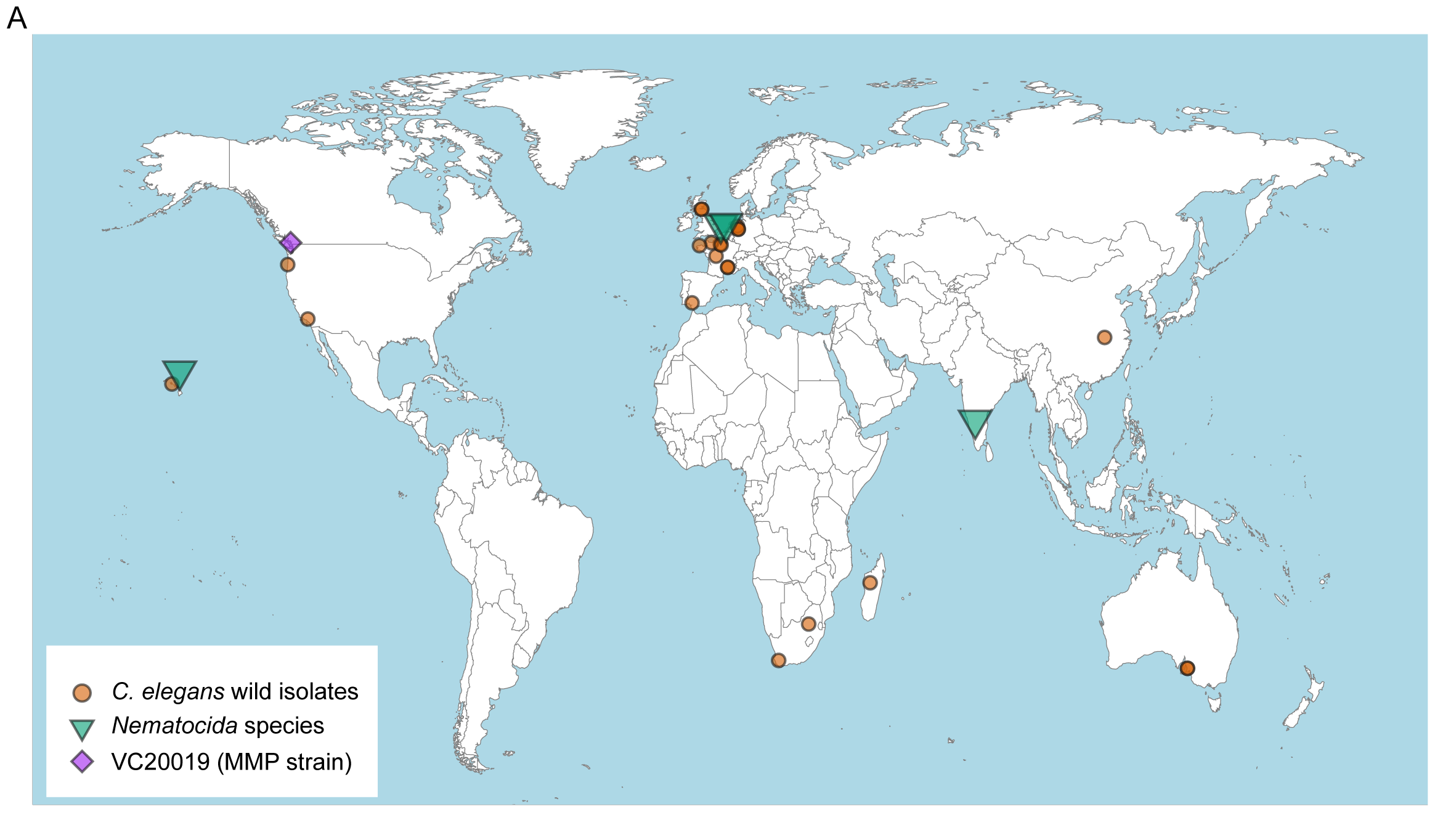

Supplement: S1 Fig — (A) World map highlighting the locations where the C. elegans wild isolate strains (orange circles), Nematocida species (green triangles), and the VC20019 reference strain (purple star) used in this study were originally isolated. Map of the world is from ggplot2 version 3.3.5. https://rdrr.io/cran/maps/man/world.html. (TIF) [file ppat.1011225.s001.tif]

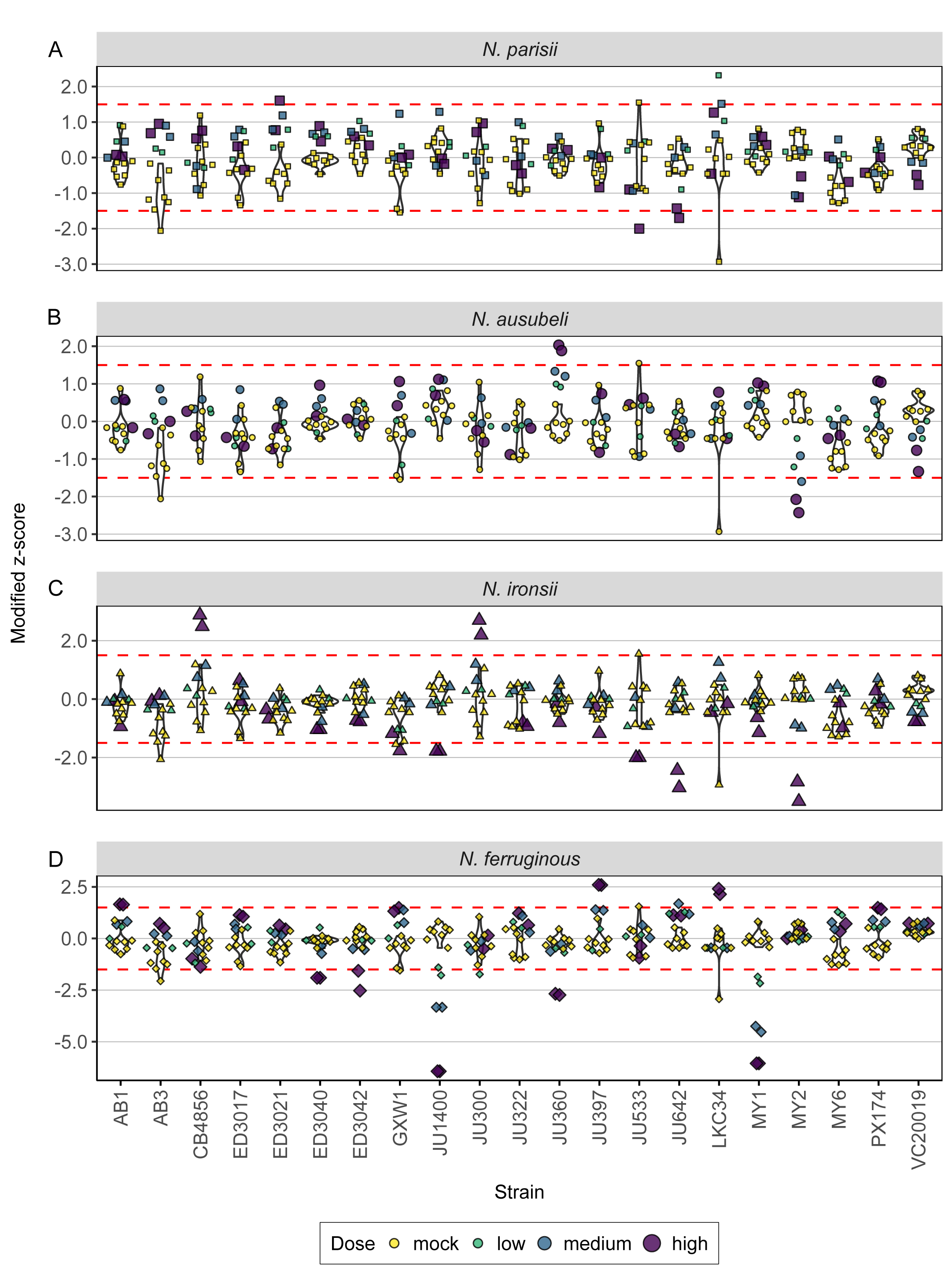

Supplement: S2 Fig — Violin plots of 20 wild isolates and lab reference-derived control strain VC20019 showing modified z-scores of mean fold-change rate (FCR) per generation. Violin shapes are derived from the eight mock-infection replicates. Overlayed are the eight mock infections replicates and six infection replicates for each of N. parisii (A), N. ausubeli (B), N. ironsii (C), and N. ferruginous (D). Replicates are coloured by mock infection (yellow), low dose (green), medium dose (blue), and high dose (purple) with increasing point size associated with increasing dose. Dotted lines represent the z-score candidate threshold of ±1.5. The strains CB4854 and JU312 were excluded from analysis due to low initial population abundance. (TIF) [file ppat.1011225.s002.tif]

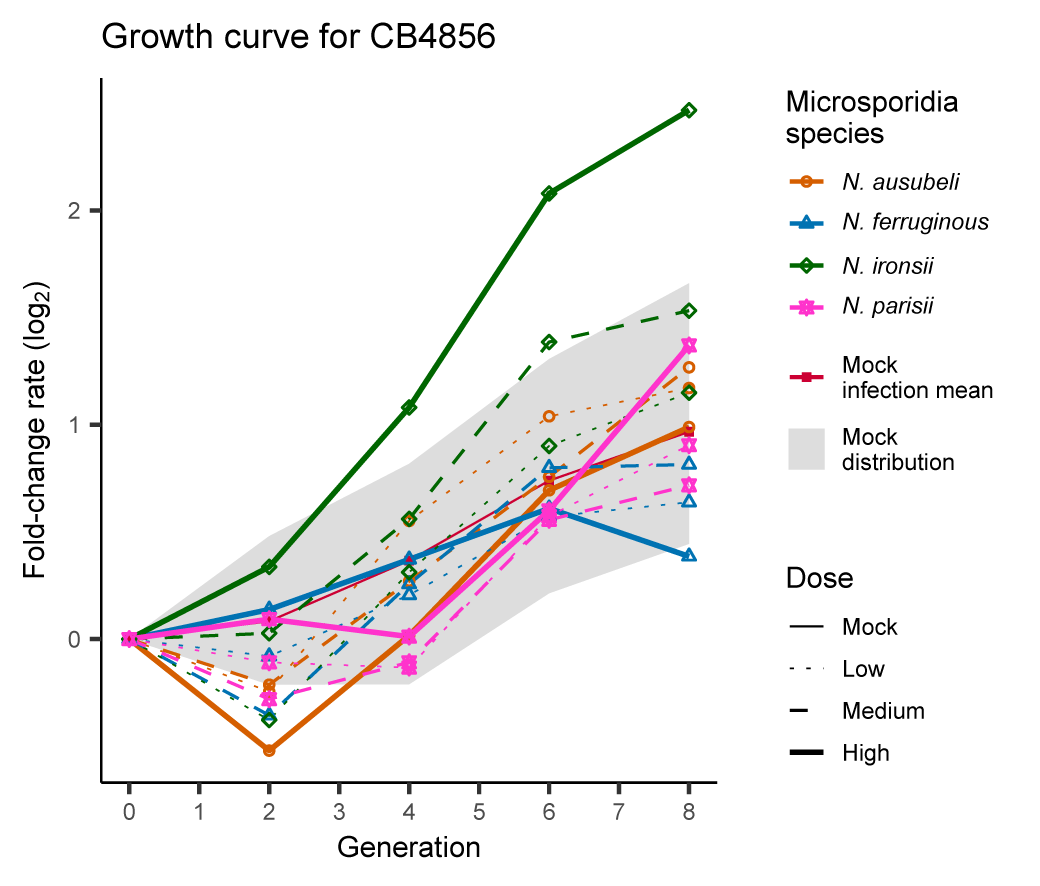

Supplement: S3 Fig — Line graph of fold change across multiple time points and conditions for CB4856 demonstrates potential fitness increase relative to mock-infection conditions when infected with a high dose of N. ironsii. Microsporidia strains are denoted by point shape and doses of microsporidia are denoted by line type. The eight uninfected control replicates are bounded within the greyed area with the mean of the uninfected controls denoted by a solid red line. All other lines represent mean FCR of two biological replicate samples. (TIF) [file ppat.1011225.s003.tif]

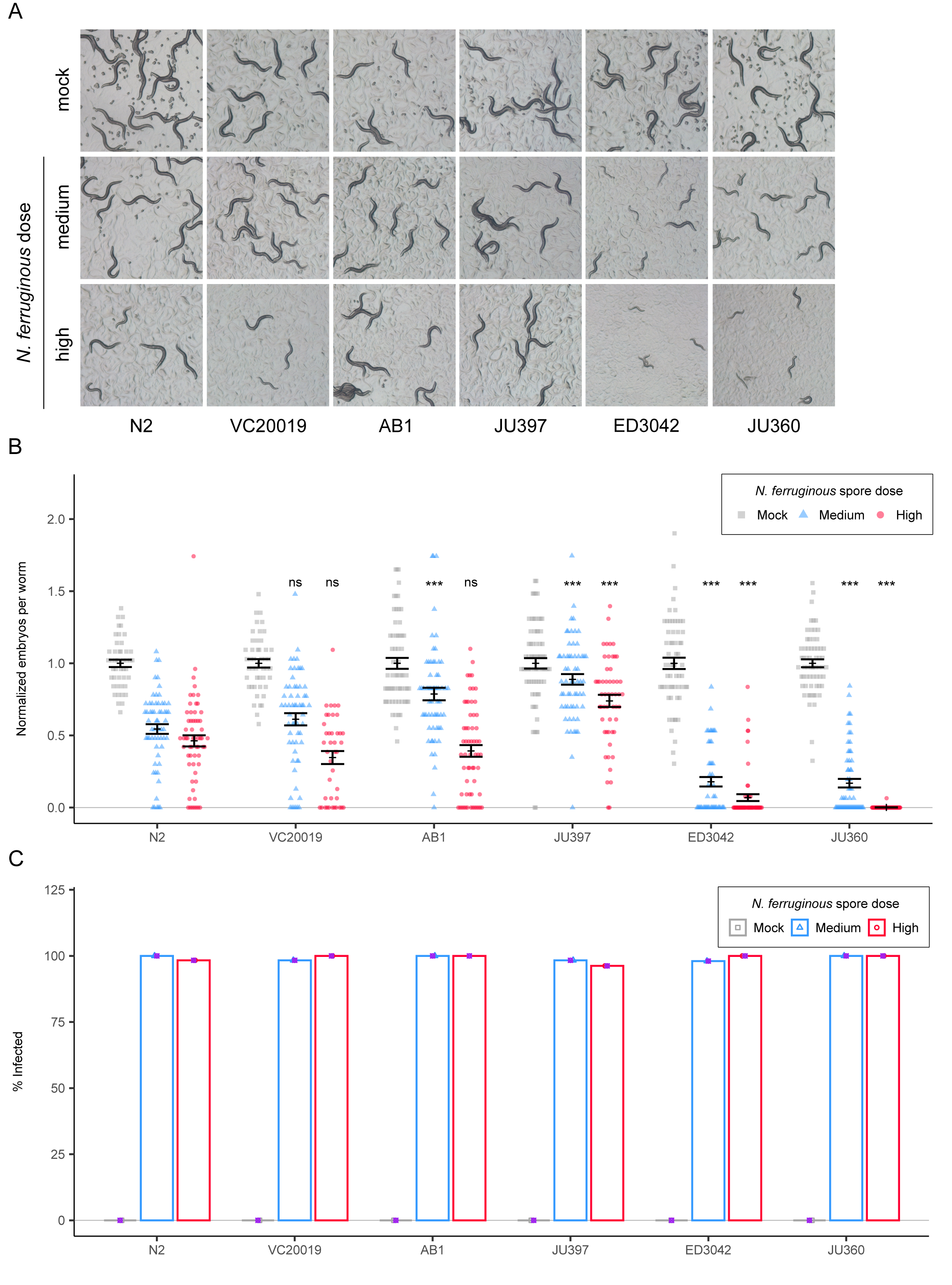

Supplement: S4 Fig — (A-C) Strains of C. elegans were infected with medium and high doses of N. ferruginous for 72 hours, imaged, fixed, and stained with DY96 and a N. ferruginous 18S rRNA FISH probe. (A) images of N2 and VC20019 controls versus AB1, JU397, ED3042, and JU360 at 72 hpi. (B) Dot plot with mean and SEM bars of normalized embryo counts from infected populations with n = {41,70} worms per sample. (C) Bar plot depicting the percent of animals with detectable meront staining. p-values denote comparison to N2 controls and were determined by two-way ANOVA with Tukey post-hoc. Significance was defined as p ≤ 0.001 (***) and not significant as p > 0.05 (ns). (TIF) [file ppat.1011225.s004.tif]

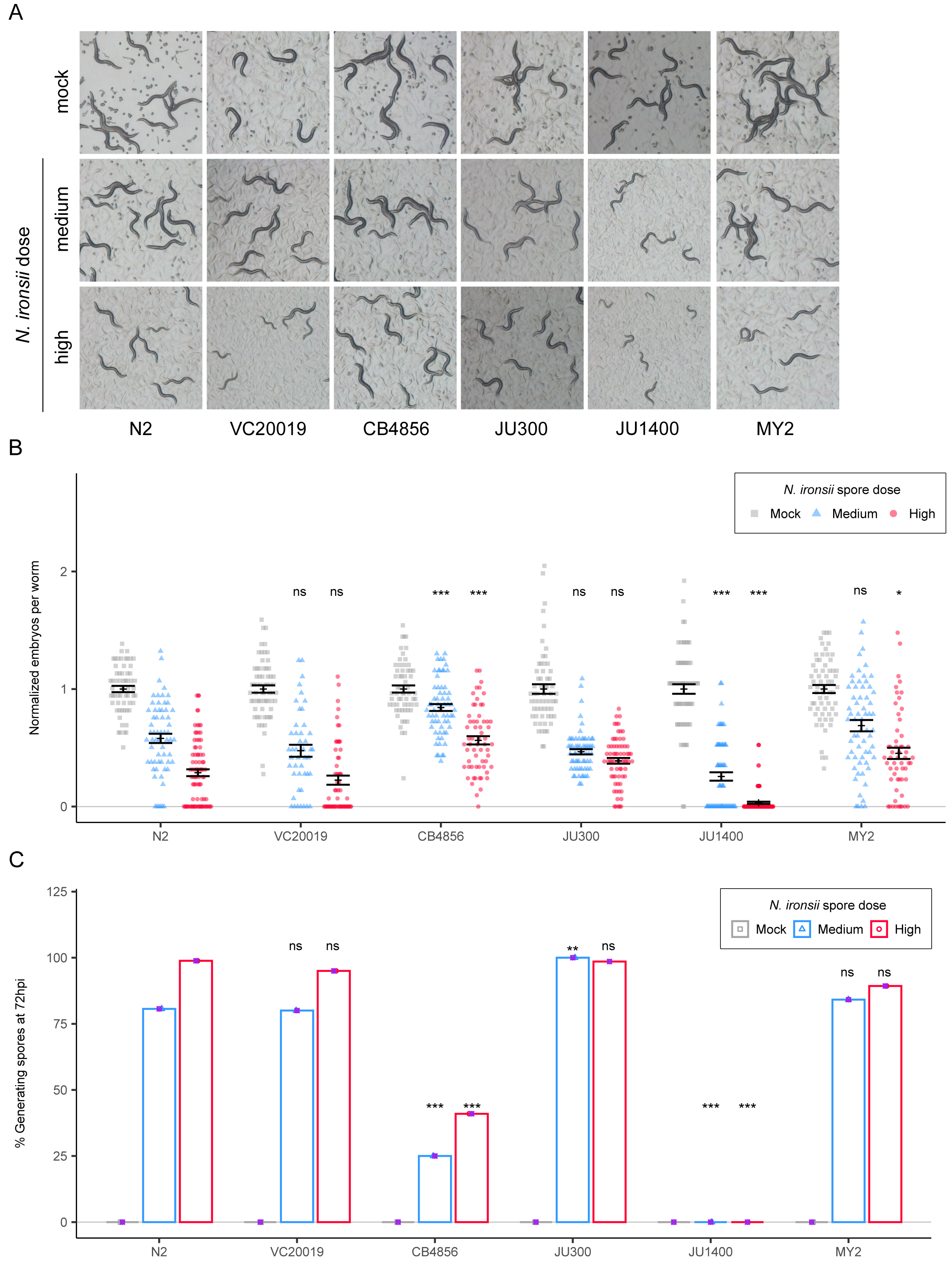

Supplement: S5 Fig — (A-C) Strains of C. elegans were infected with medium and high doses of N. ironsii for 72 hours, imaged, fixed, and stained with DY96. (A) images of N2 and VC20019 controls versus CB4856, JU300, JU1400, and MY2 at 72 hpi. (B) Dot plot with mean and SEM bars of normalized embryo counts from infected populations with n = {45,115} worms per sample. (C) Bar plot depicting the percent of animals with newly generated spores. p-values denote comparison to N2 controls and were determined by two-way ANOVA with Tukey post-hoc. Significance was defined as p ≤ 0.001 (***), p ≤ 0.01 (**), p ≤ 0.05 (*) and not significant as p > 0.05 (ns). (TIF) [file ppat.1011225.s005.tif]

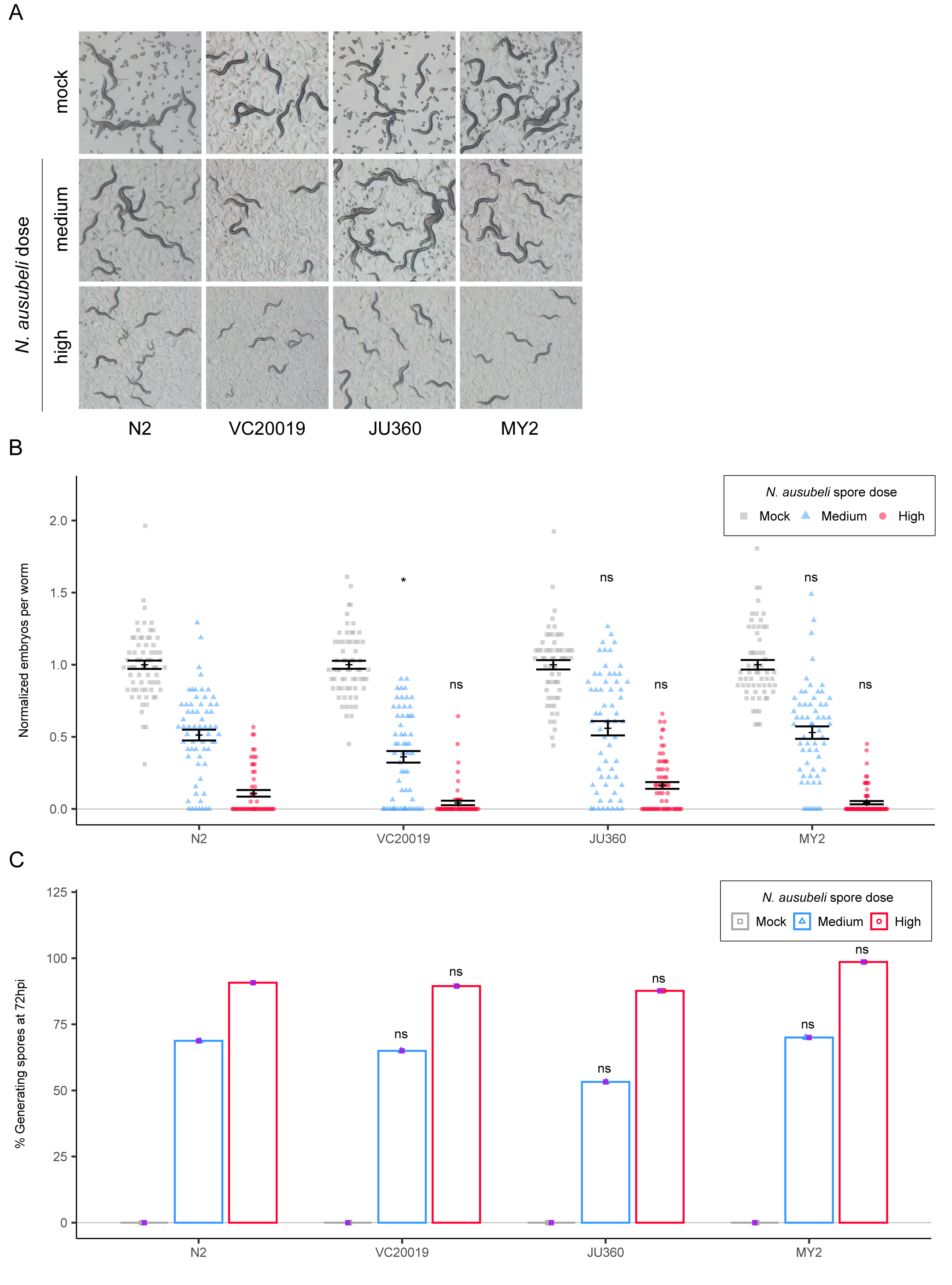

Supplement: S6 Fig — (A-C) Strains of C. elegans were infected with medium and high doses of N. ausubeli for 72 hours, imaged, fixed, and stained with DY96. (A) images of N2 and VC20019 controls versus JU360 and MY2 at 72 hpi. (B) Dot plot with mean and SEM bars of normalized embryo counts from infected populations with n = {54,71} worms per sample. (C) Bar plot depicting the percent of animals with newly generated spores. p-values denote comparison to N2 controls and were determined by two-way ANOVA with Tukey post-hoc. Significance was defined as p ≤ 0.05 (*) and not significant as p > 0.05 (ns). (TIF) [file ppat.1011225.s006.tif]

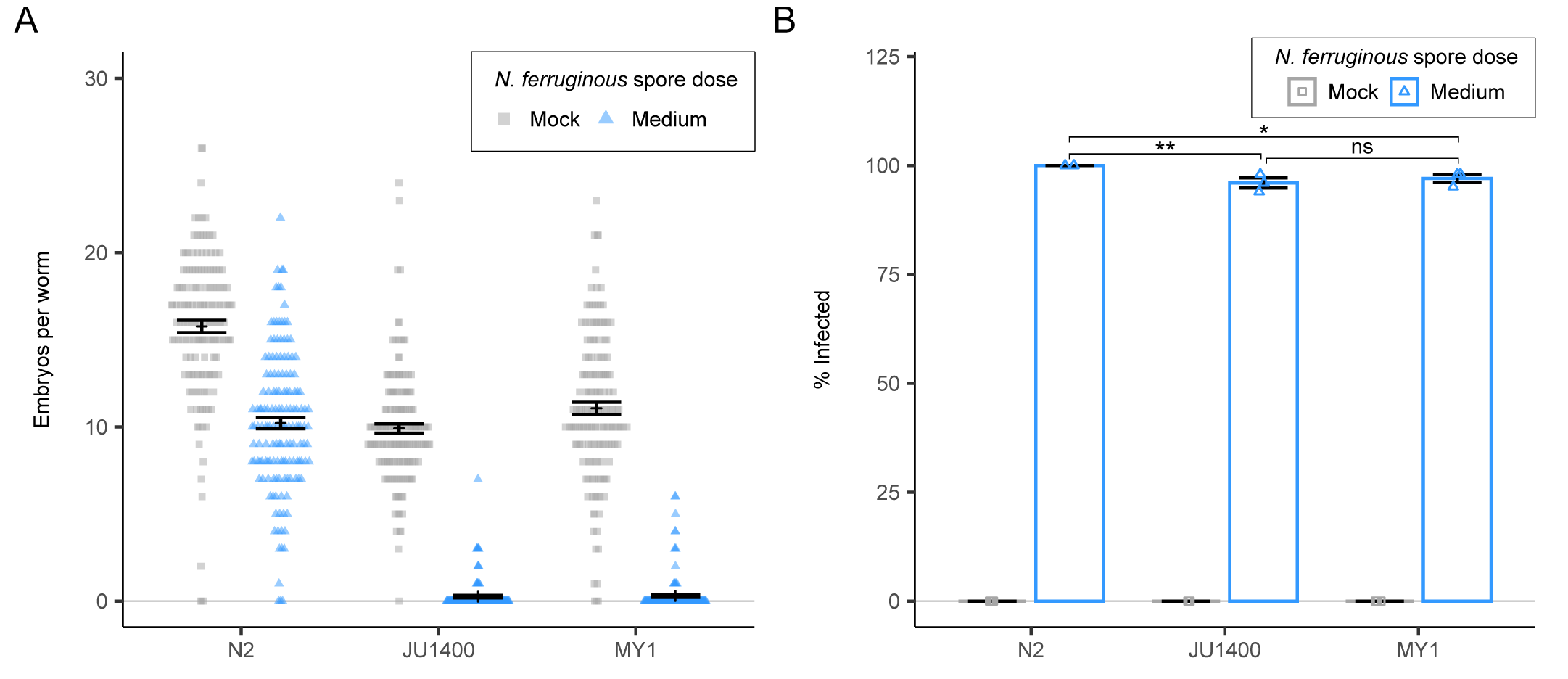

Supplement: S7 Fig — JU1400, MY1, and N2 L1s were infected with a medium dose of N. ferruginous for 72 hours, fixed, and stained with N. ferruginous 18S rRNA FISH probes and DY96. (A) Dot plot with mean and SEM bars of embryo counts from infected populations. (B) Bar plot with SEM bars depicting the percent of animals with visible meronts by FISH staining. Data is combined from three biological replicates with n = {41,50} worms per replicate sample. p-values denote comparison to N2 controls and were determined by two-way ANOVA with Tukey post-hoc. Significance was defined as p ≤ 0.01 (**), p ≤ 0.05 (*) and not significant as p > 0.05 (ns). (TIF) [file ppat.1011225.s007.tif]

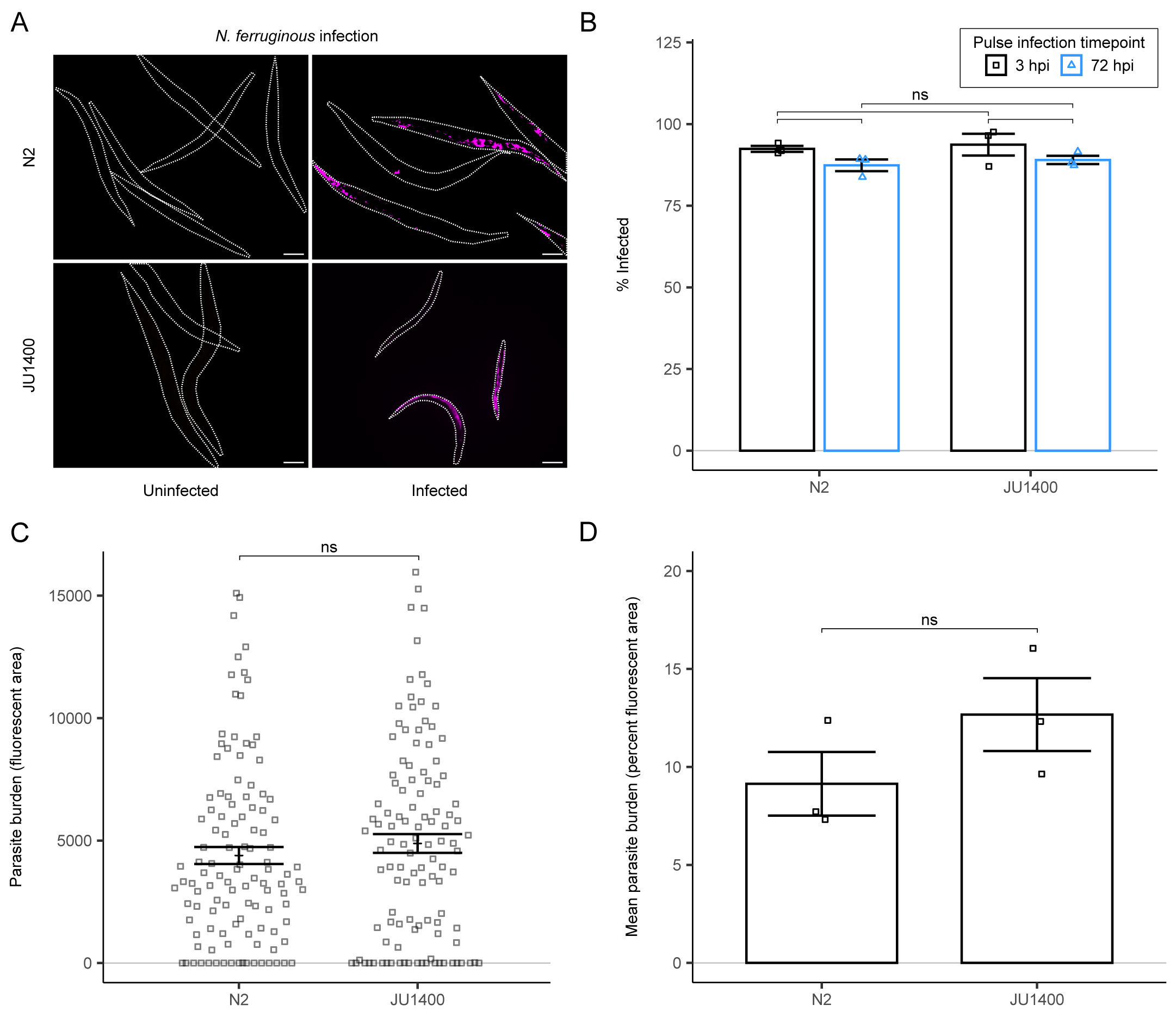

Supplement: S8 Fig — (A-D) JU1400 and N2 L1s were infected with 1.25 million spores of N. ferruginous for 3 h, washed to remove spores, and replated for an additional 69 h. Animals were fixed at 3 hpi and 72 hpi and stained with N. ferruginous 18S rRNA FISH probes. (A) Representative images of infected animals at 72 hpi. Scale bars are 100 μm. (B) Bar plot with SEM bars depicting the percent of animals displaying either sporoplasm (3 hpi) or meronts (72 hpi) by FISH staining. (C-D) quantitation of FISH stain at 72 hpi. (C) Dot plot with mean and SEM bars of parasite burden by area in infected populations with a minimum of n = {41,50} worms per sample. (D) Bar plot with SEM bars depicting mean parasite burden as a function of percent fluorescent area per animal by FISH staining. Data in (C, D) is combined from three biological replicates with n = 40 worms per replicate sample. p-values denote comparison to N2 or JU1400 controls and were determined by two-way ANOVA with Tukey post-hoc. Not significant was defined as p > 0.05 (ns). (TIF) [file ppat.1011225.s008.tif]

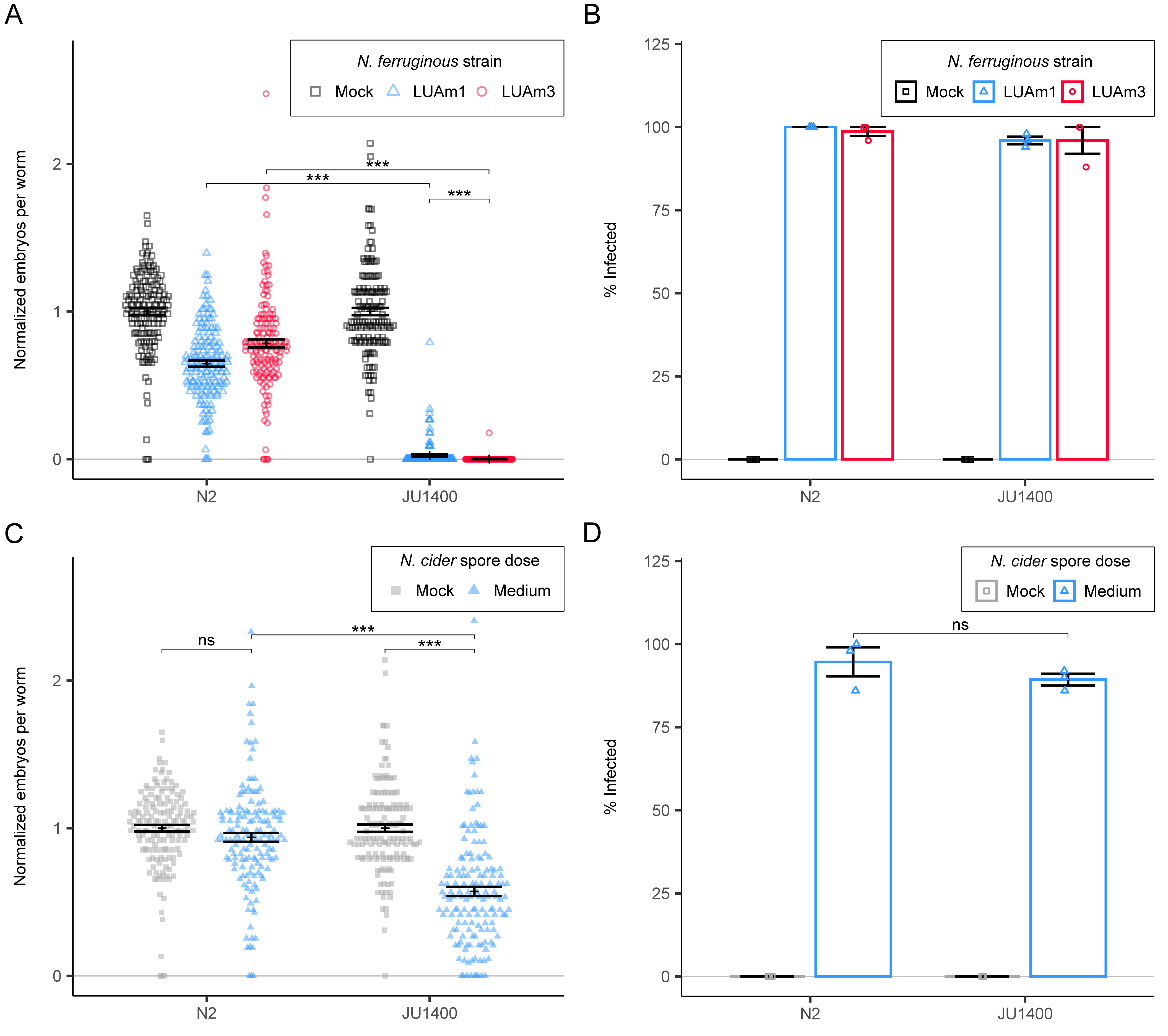

Supplement: S9 Fig — (A-D) N2 and JU1400 L1 stage animals were infected with medium doses of N. ferruginous strain LUAm1 or LUAm3 (A-B) or a medium dose of N. cider (C-D) for 72 hours, fixed, and stained with DY96 and 18S rRNA FISH probe. (A, C) Dot plot of normalized embryo counts of animals with mean and SEM bars. (B, D) Bar plot with SEM bars of percent population with meronts visible. Data are combined from three biological replicates with n = 50 worms per replicate. p-values were determined by two-way ANOVA with Tukey post-hoc. Significance was defined as p ≤ 0.05 (*), p ≤ 0.001 (***), and not significant as p > 0.05 (ns). (TIF) [file ppat.1011225.s009.tif]

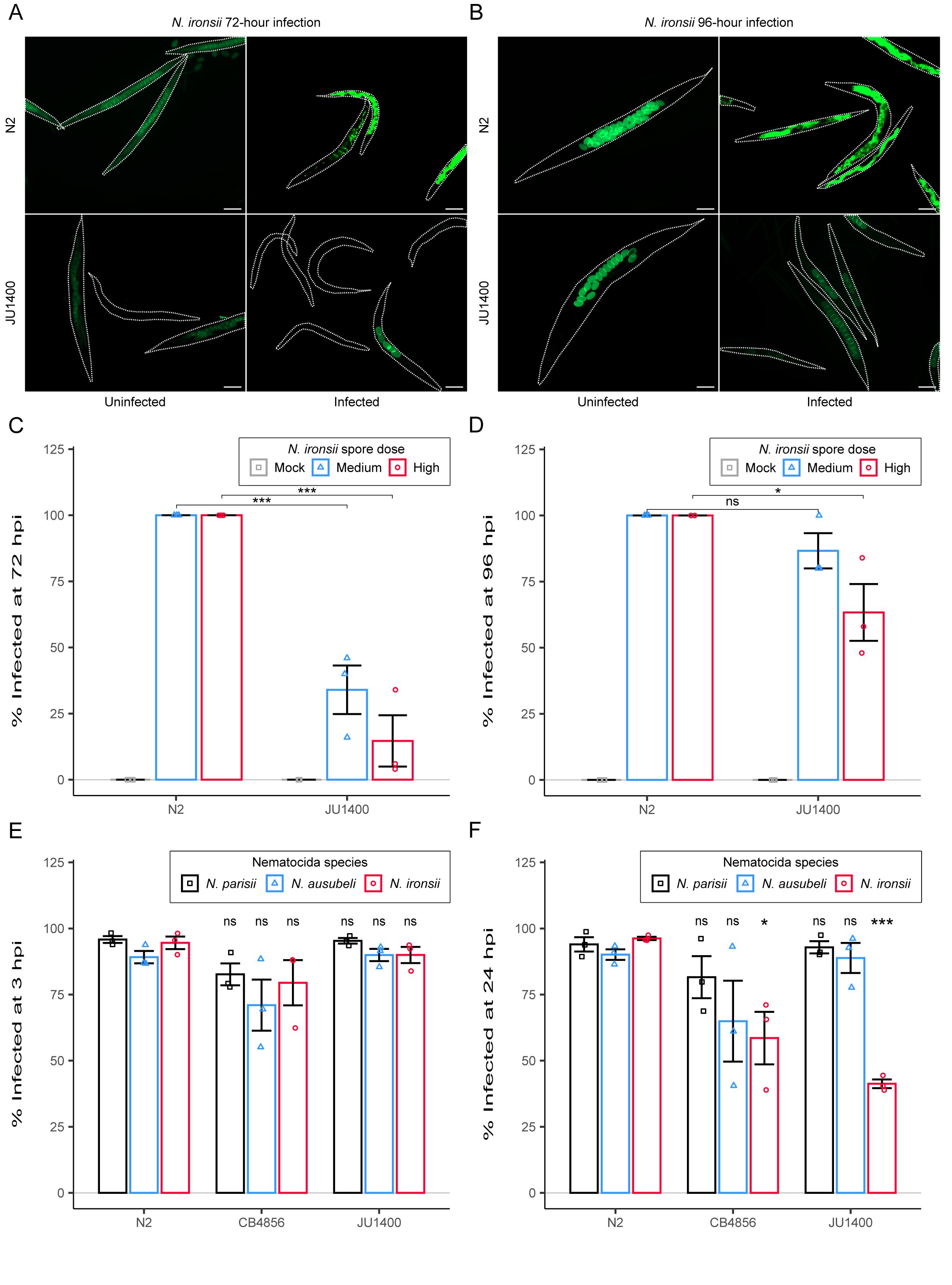

Supplement: S10 Fig — N2 and JU1400 L1 stage animals were infected with mock, medium, and high doses of N. ironsii for 72 (A and C) and 96 (B and D) hours and fixed. Embryos and mature spores are visualized by staining with DY96 (green), meronts are stained by a 18S rRNA FISH probe. (A-B) Representative images of high dose infection from each experiment. Scale bars are 100 μm. (C-D) Bar plots with SEM bars of percent population with meronts visible. Data in (C, D) is combined from three biological replicates each with n = {47,50} worms per replicate. (E-F) N2, JU1400, and CB4856 animals at the L1 stage were infected for 3 hours with 1.25 million spores of either N. parisii, N. ausubeli, or N. ironsii before washing away excess spores. Animals were either fixed at 3 hours (E) or replated for an additional 21 hours (F) and then fixed. Shown are bar plots with SEM bars of percent population with meronts visible. Data in (E, F) are combined from three biological replicates each with n = {59,179} worms per replicate. p-values were determined by two-way ANOVA with Tukey post-hoc. Significance was defined as p ≤ 0.05 (*), p ≤ 0.001 (***), and not significant as p > 0.05 (ns). (TIF) [file ppat.1011225.s010.tif]

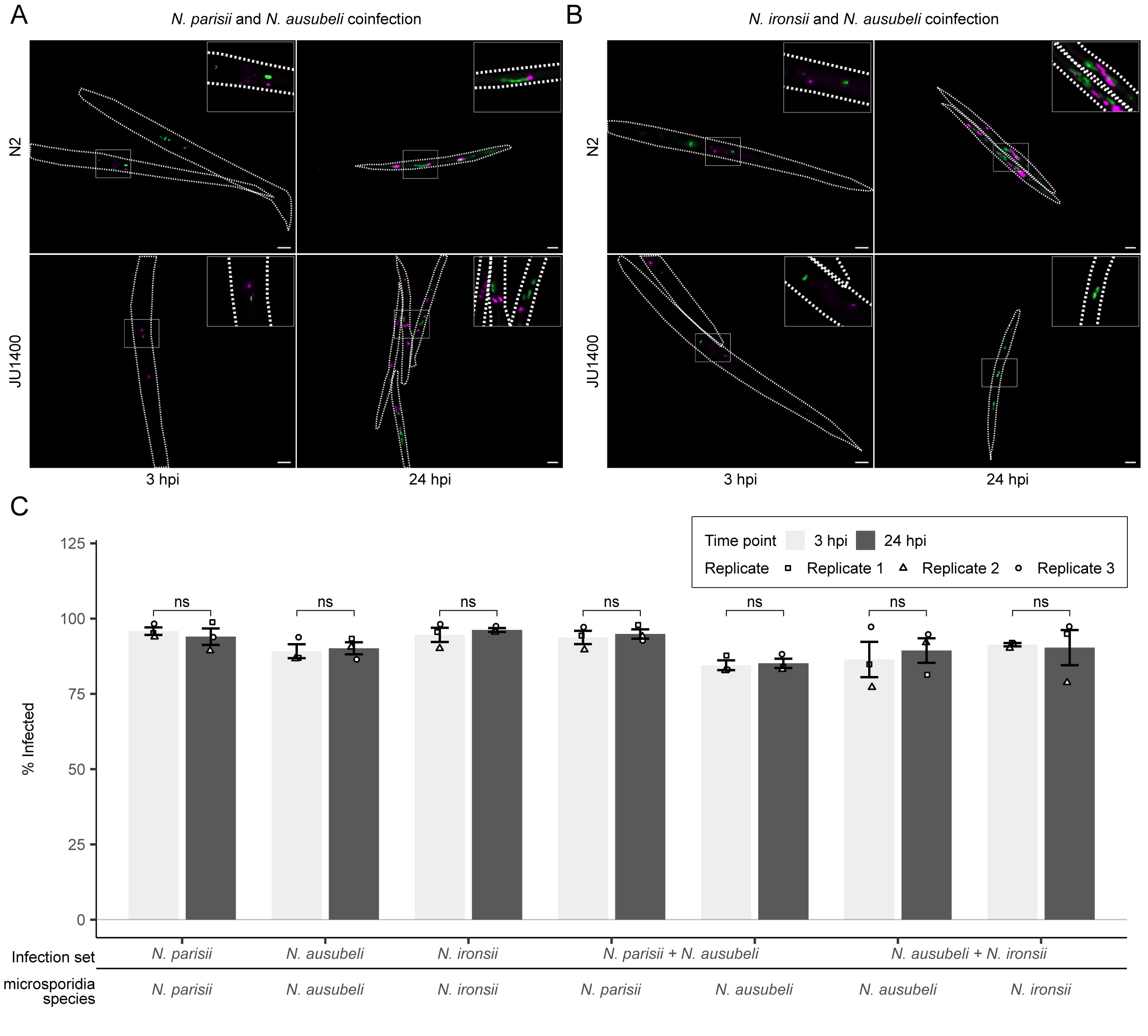

Supplement: S11 Fig — N2 and JU1400 animals at the L1 stage were pulse infected for 3 hours with N. parisii, N. ausubeli, N. ironsii, N. parisii + N. ausubeli, or N. ausubeli + N. ironsii before washing away excess spores. Animals were either fixed immediately at 3 hpi or at 24 hpi. Samples were then stained with species-specific 18S RNA FISH probes (N. ausubeli = green; N. parisii and N. ironsii = magenta) and animals containing either sporoplasms or meronts were counted as infected. (A) Representative images of N2 and JU1400 infected with N. parisii and N. ausubeli (A) and N. ironsii and N. ausubeli (B). Scale bar 10 μm for 3 hpi and 25 μm for 24 hpi. Insets at top right for each image show an enlarged image of the outlined boxes. (C) Bar plot with SEM bars show percent of N2 animals infected per replicate from three biological replicates with n = {59, 179} worms per replicate. p-values were determined by two-way ANOVA with Tukey post-hoc. Non-significance was defined as p > 0.05 (ns). (TIF) [file ppat.1011225.s011.tif]

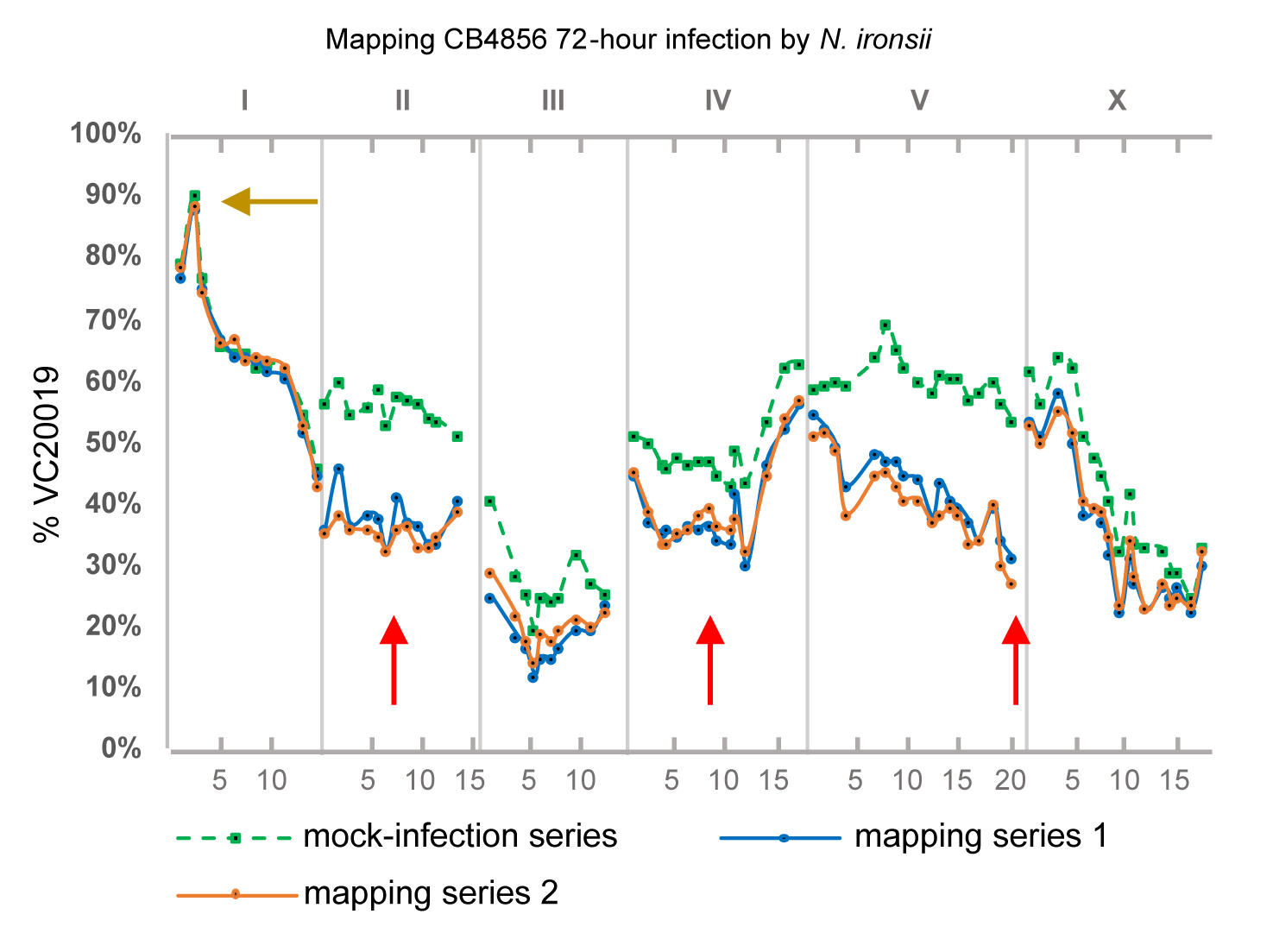

Supplement: S12 Fig — Genetic mapping of CB4856 resistance to N. ironsii was performed with MIP-MAP using the AWR133 mapping strain. Green square-points and dotted lines represent a mock-infection replicate with the solid blue and orange lines representing two biological replicates exposed to high dose infection conditions for 72-hours per selection cycle. A red arrow on chromosome V indicates a strong signal on the right arm. Additional red arrows on chromosomes II and IV indicate broad regions of interest across the chromosomes that may contribute to CB4856 resistance phenotypes. Golden arrow indicates the zeel-1/peel-1 incompatibility locus at chromosome I. (TIF) [file ppat.1011225.s012.tif]

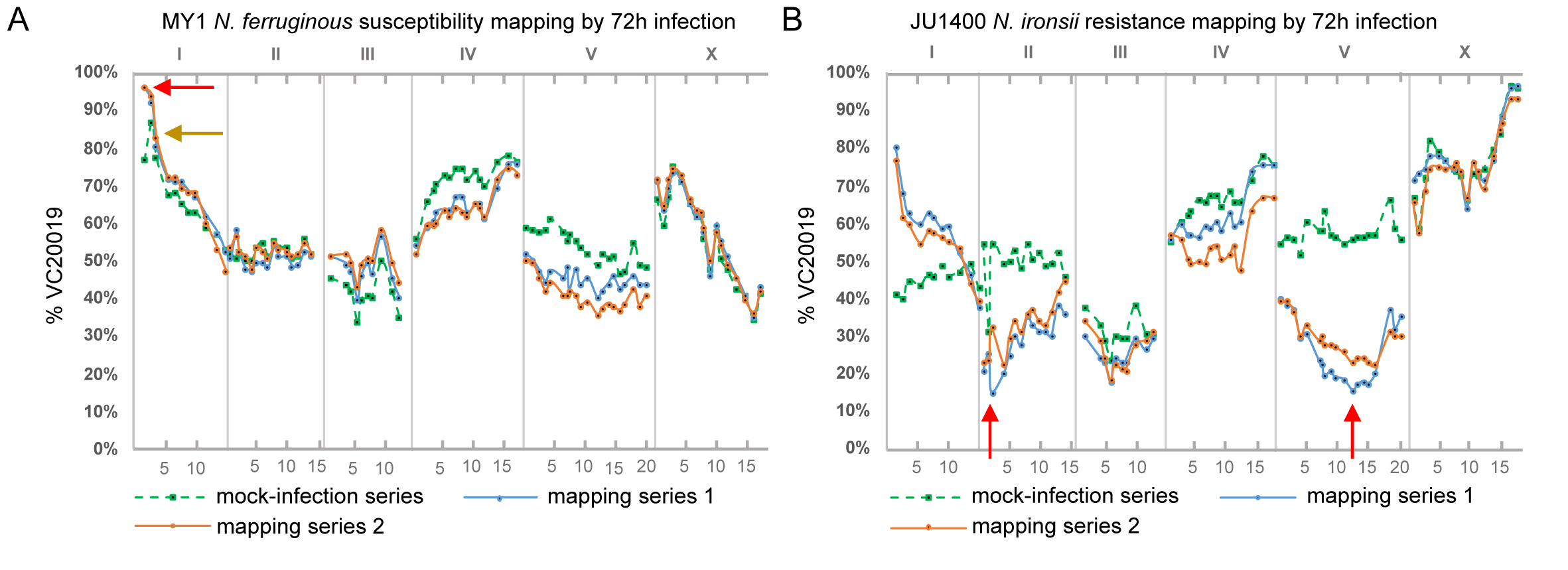

Supplement: S13 Fig — Genetic mapping of (A) MY1 susceptibility to N. ferruginous and (B) JU1400 resistance to N. ironsii was performed with MIP-MAP using the AWR133 mapping strain. Green square-points and dotted lines represent a mock-infection replicate with the solid blue and orange lines representing two biological replicates exposed to specific infection conditions. Red arrows indicate candidate regions of interest based on the expected genome fixation direction during phenotype selection. Golden arrow indicates the zeel-1/peel-1 incompatibility locus at chromosome I. (TIF) [file ppat.1011225.s013.tif]

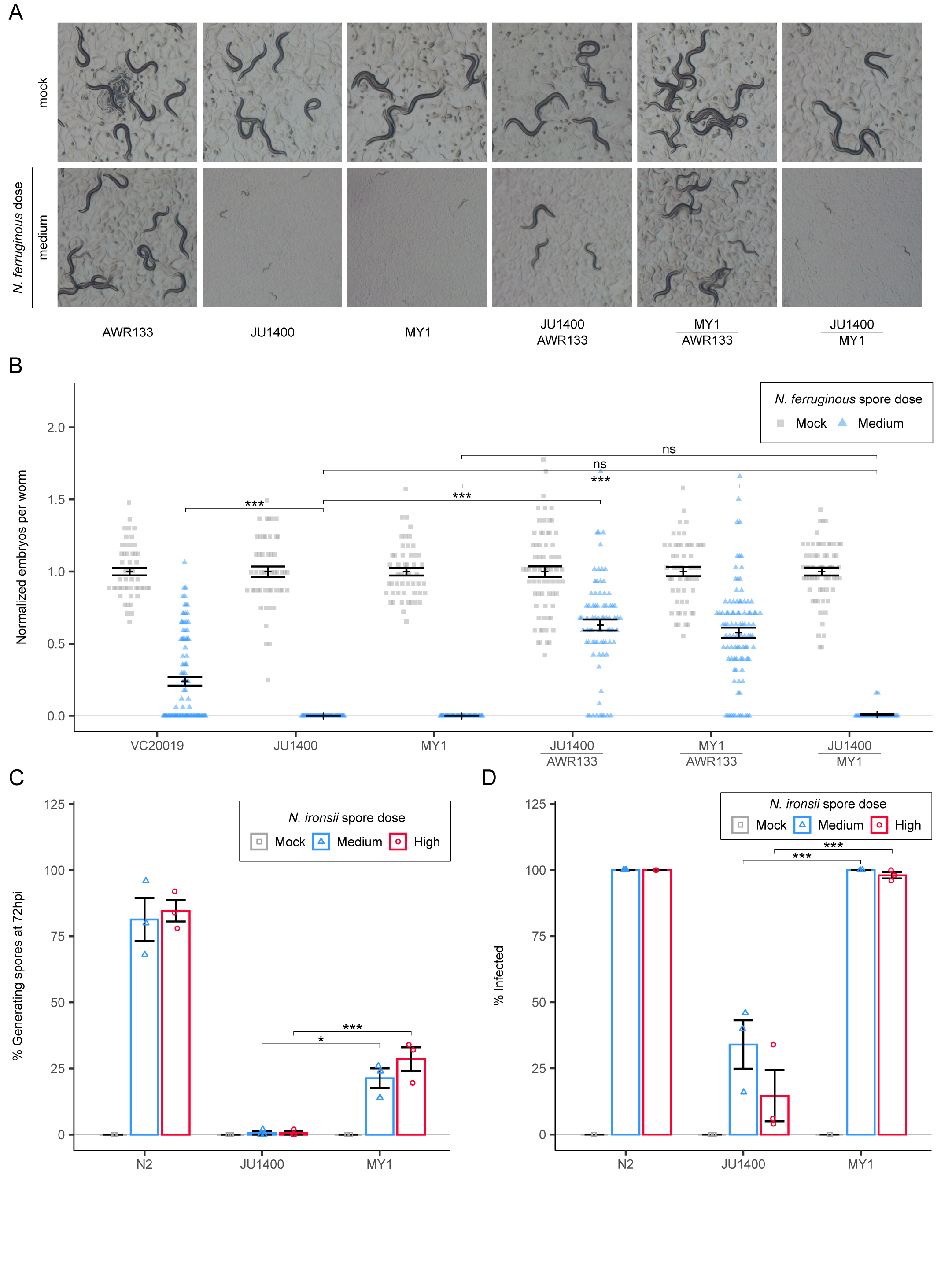

Supplement: S14 Fig — (A-B) Analysis of F1 progeny from crosses between AWR133 (VC20019 with integrated pmyo-2::GFP) and the wild isolates JU1400 or MY1 as well as F1 progeny from crosses between JU1400 hermaphrodites and MY1 males. F1s were examined 72 hours post-infection by N. ferruginous for body size (A) and normalized embryo counts (B). (B) Dot plot with mean and SEM bars of normalized embryo counts from infected populations with a minimum of n = {47,51} worms per sample. (C-D) L1 stage animals were infected for 72 hours with N. ironsii, fixed, and stained with DY96 and an 18S rRNA FISH probe. Bar plots depict the percent of animals with newly generated spores (C) or meront signal (D). p-values were determined by two-way ANOVA with Tukey post-hoc. Significance was defined as p ≤ 0.001 (***), p ≤ 0.05 (*) and not significant as p > 0.05 (ns). (TIF) [file ppat.1011225.s014.tif]

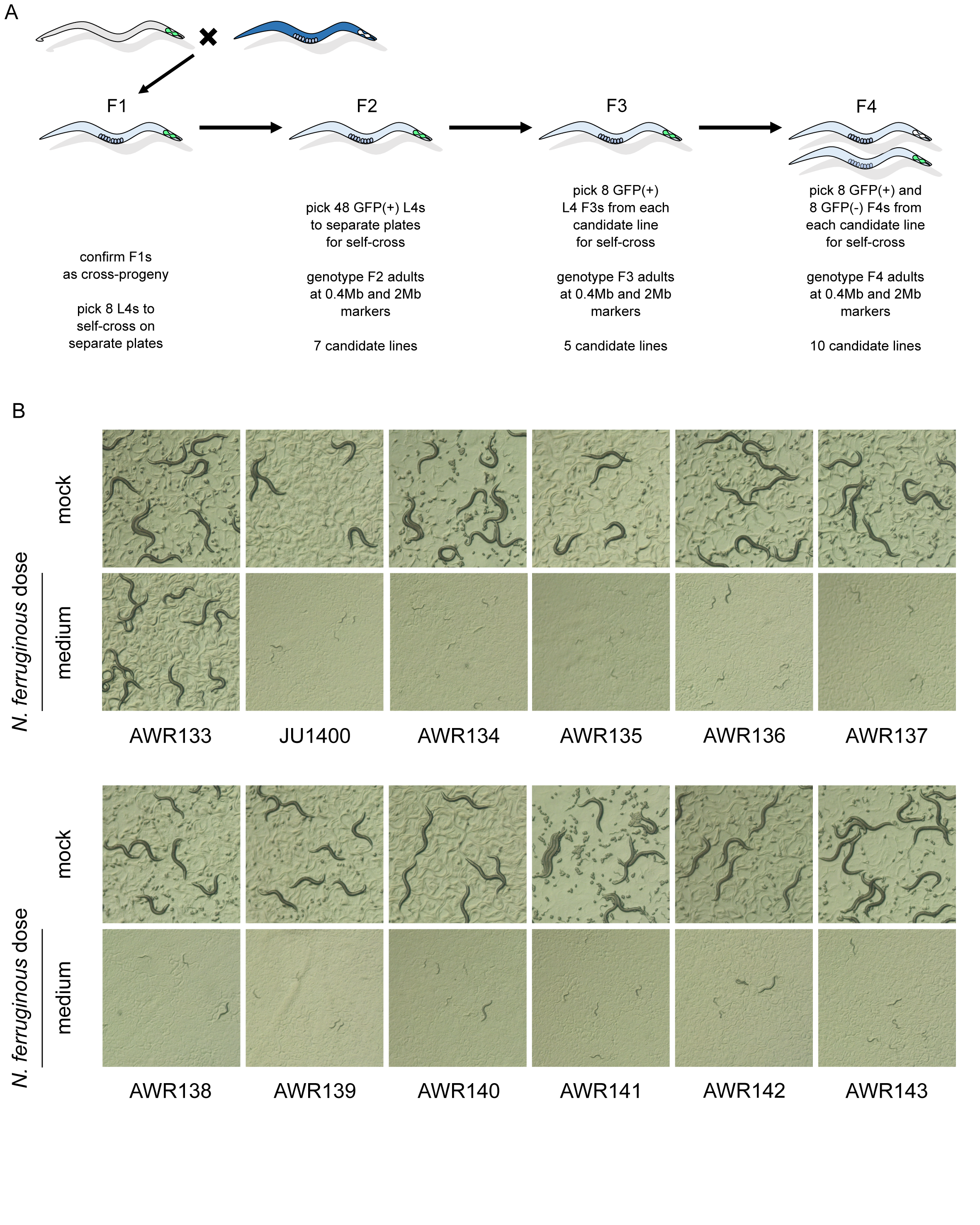

Supplement: S15 Fig — A schematic (A) of 10 separate NILs generated through a cross between AWR133 and JU1400. These lines were infected with a medium dose of N. ferruginous for 72 hours (B) and their resulting body size phenotype was assessed in comparison to N2 and JU1400 control strains. (TIF) [file ppat.1011225.s015.tif]

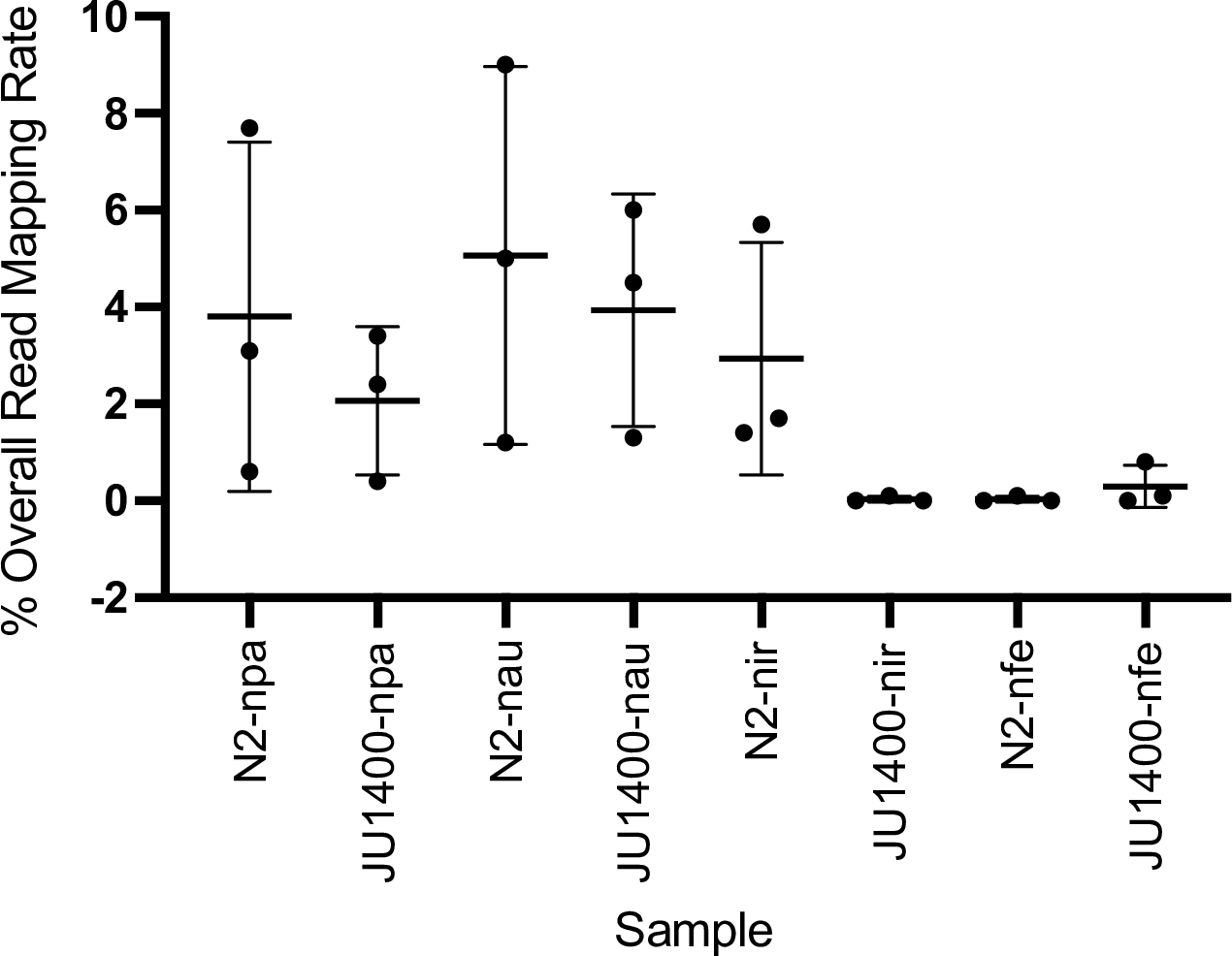

Supplement: S16 Fig — Scatterplot of overall read mapping rate of Nematocida in both strains with mean and standard deviation. uni (uninfected), npa (N. parisii), nau (N. ausubeli), nir (N. ironsii), and nfe (N. ferruginous). (TIF) [file ppat.1011225.s016.tif]

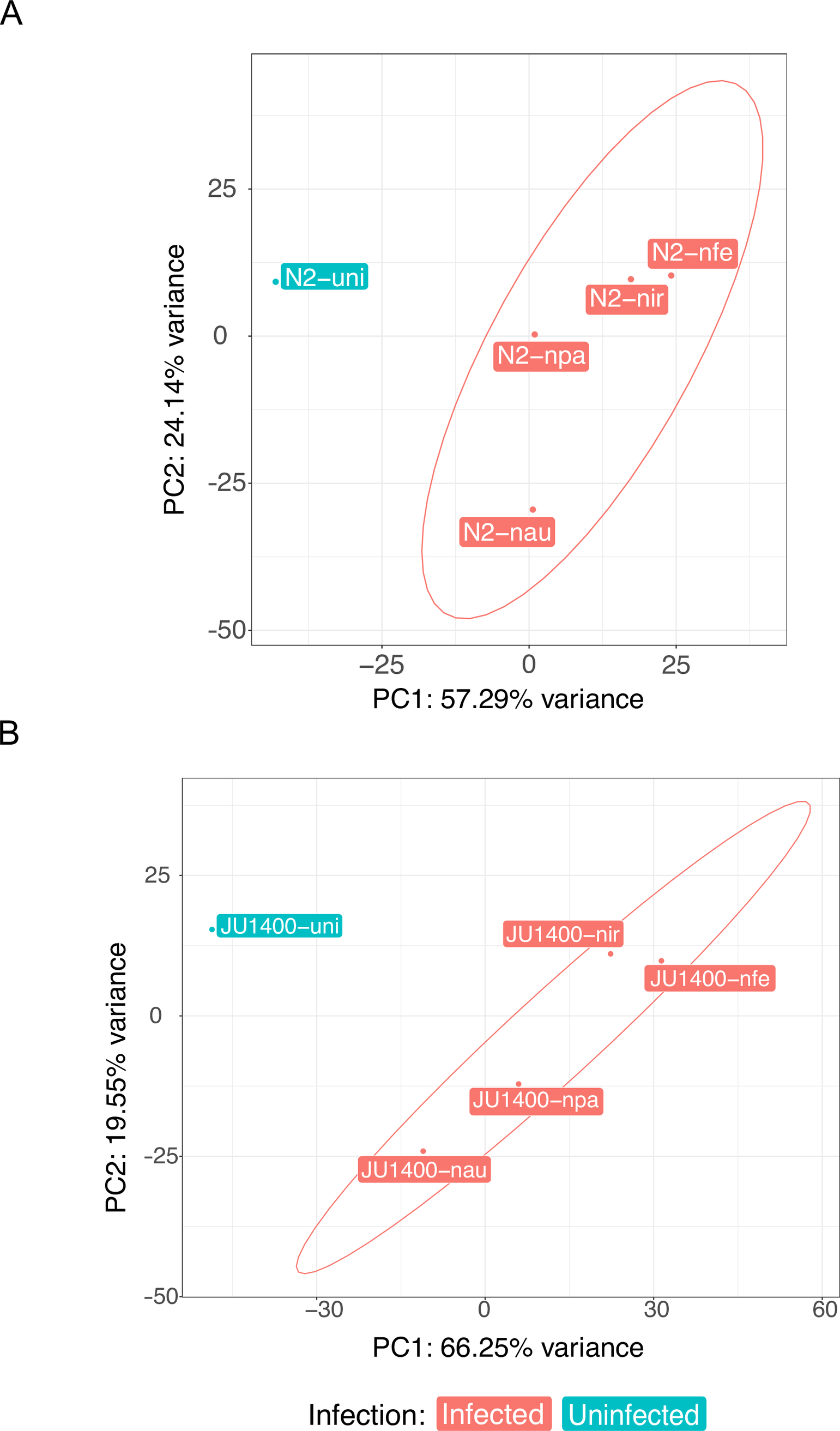

Supplement: S17 Fig — Principal component plots of expression data from only (A) N2 samples or (B) JU1400 samples. Circles represent confidence ellipses around each strain at 95% confidence interval. uni (uninfected), npa (N. parisii), nau (N. ausubeli), nir (N. ironsii), and nfe (N. ferruginous). (TIF) [file ppat.1011225.s017.tif]

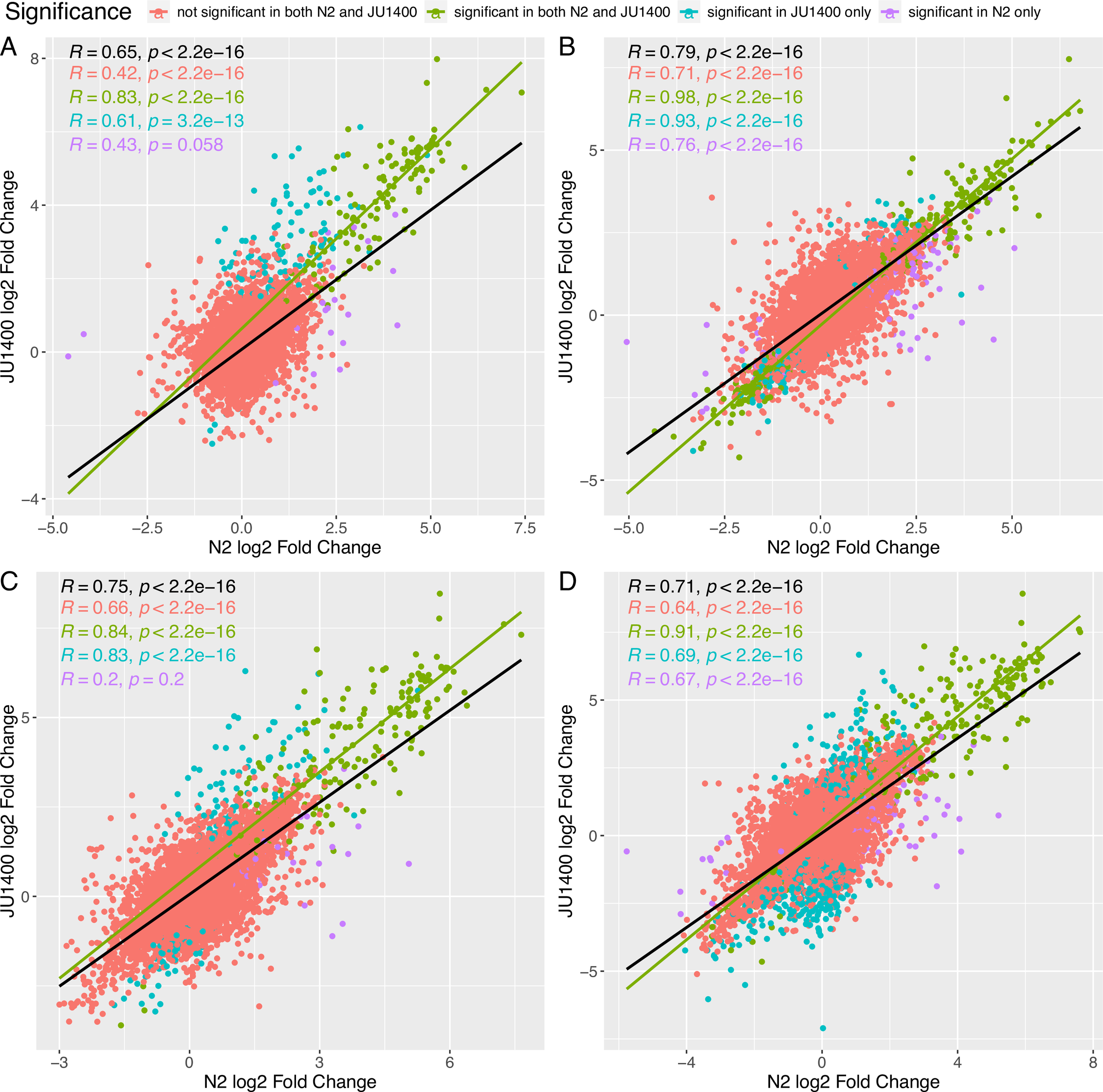

Supplement: S18 Fig — Correlation between log2 fold changes in genes of N2 and JU1400 animals infected with (A) N. parisii (B) N. ausubeli (C) N. ironsii (D) N. ferruginous. Pearson’s correlation value and p-value for each cluster (pink, green, blue, purple) are presented in the upper left corner. Pearson’s correlation value and p-value for all the genes are presented in the bottom left corner. A gene with FDR adjusted p-value of <0.01 is deemed significant in each strain. (TIF) [file ppat.1011225.s018.tif]

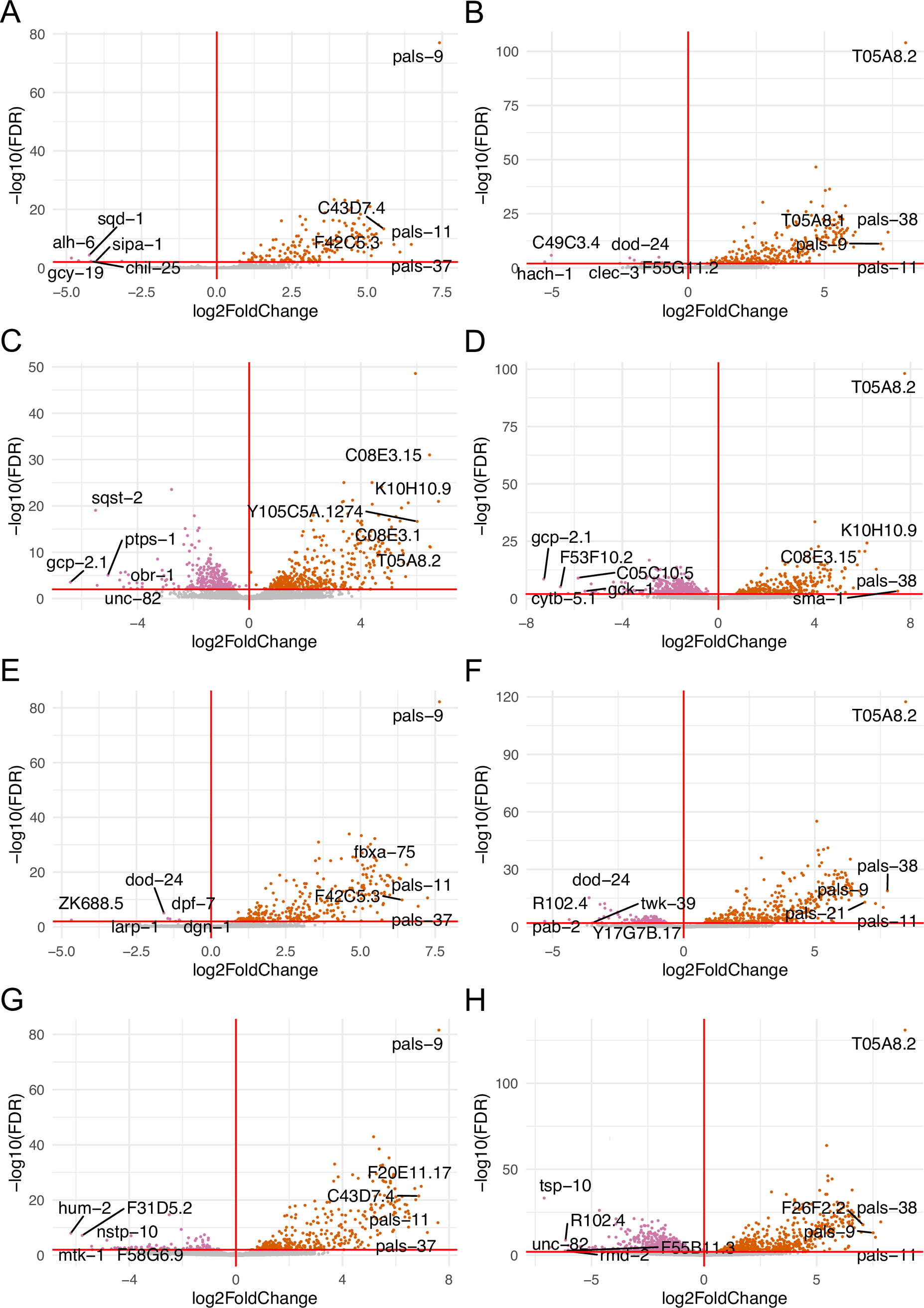

Supplement: S19 Fig — (A) N. parisii-infected N2. (B) N. parisii-infected JU1400. (C) N. ausubeli-infected N2. (D) N. ausubeli-infected JU1400. (E) N. ironsii-infected N2. (F) N. ironsii-infected JU1400. (G) N. ferruginous-infected N2. (H) N. ferruginous-infected JU1400. Each point represents a gene, orange points indicate differentially upregulated genes, pink points are differentially downregulated genes while grey points represent genes with FDR-adjusted p-value >0.01 (red horizontal line). The top five differentially upregulated and downregulated genes with FDR-adjusted p-value <0.01 are labelled with their respective gene names. (TIF) [file ppat.1011225.s019.tif]

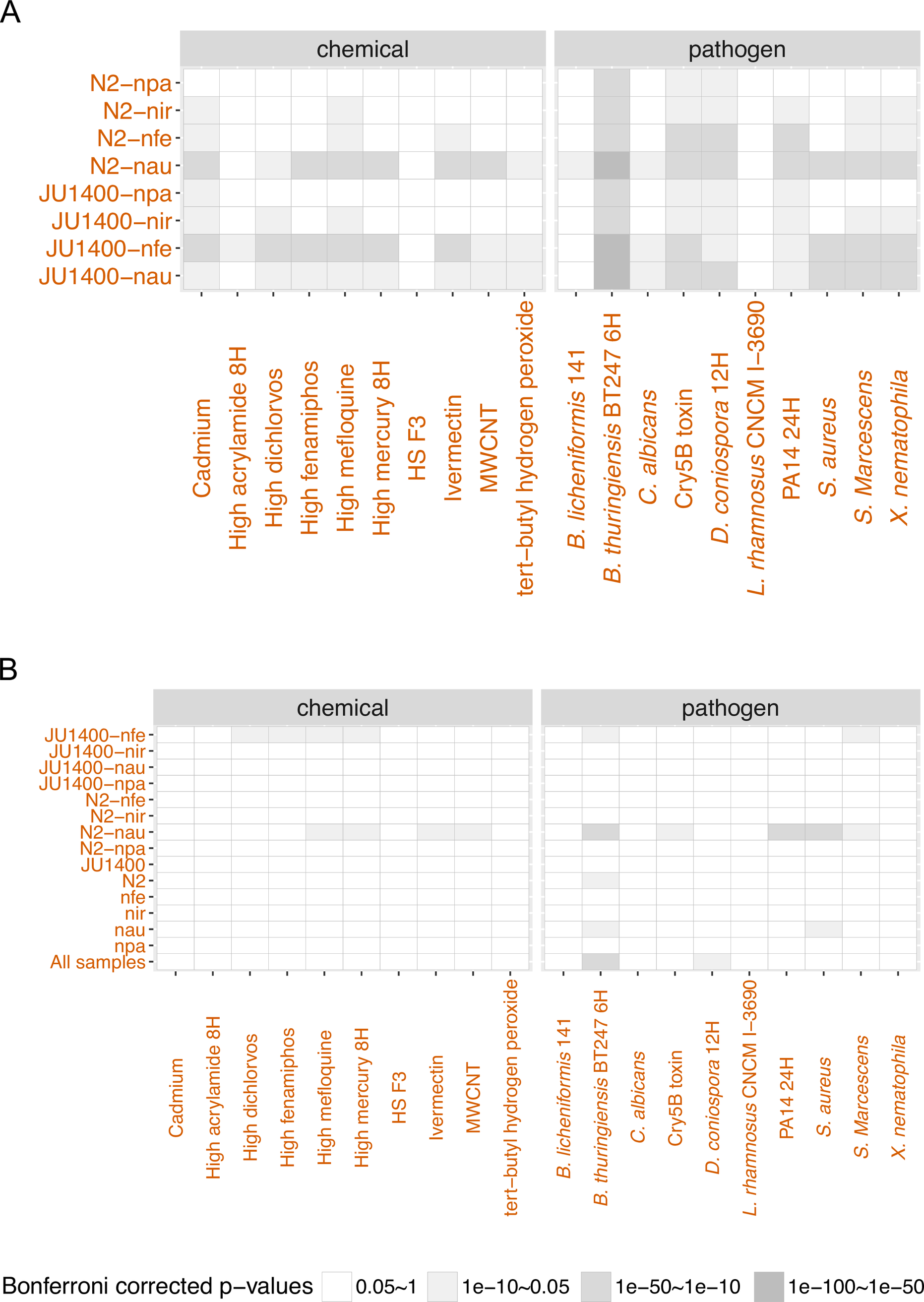

Supplement: S20 Fig — (A) upregulated genes from microsporidia-infected samples. (B) upregulated gens from strain specific, pathogen specific, and strain-pathogen specific gene sets. uni (uninfected), npa (N. parisii), nau (N. ausubeli), nir (N. ironsii), and nfe (N. ferruginous). (TIF) [file ppat.1011225.s020.tif]
