# Supplementary material for: High-throughput phenotyping of infection by diverse microsporidia species reveals a wild C. elegans strain with opposing resistance and susceptibility traits
Source: PLoS Pathog. 2023 Mar 9;19(3):e1011225. doi: 10.1371/journal.ppat.1011225 (PMC10030041; doi:10.1371/journal.ppat.1011225)
Supplement: S1 Table — (DOCX) [file ppat.1011225.s021.docx]

**Table S1. Microsporidia species information.**

| **Microsporidia species** | **Original host** | **Infected tissue** | **Geographical origin** |
| --- | --- | --- | --- |
| *Nematocida parisii* [1] | *C. elegans* | Intestine | France |
| *Nematocida ausubeli* [1,2] | *C. briggsae* | Intestine | India |
| *Nematocida ironsii* [3,4] | *C. briggsae* | Intestine | Hawaii |
| *Nematocida ferruginous* [5] | *C. elegans* | Epidermis/muscle | France |

**Reference**

1. Troemel ER, Felix M-A, Whiteman NK, Barriere A, Ausubel FM. Microsporidia are natural intracellular parasites of the nematode Caenorhabditis elegans. PLoS Biol. 2008;6: 2736–2752. doi:10.1371/journal.pbio.0060309

2. Zhang G, Sachse M, Prevost M-C, Luallen RJ, Troemel ER, Felix M-A. A Large Collection of Novel Nematode-Infecting Microsporidia and Their Diverse Interactions with Caenorhabditis elegans and Other Related Nematodes. PLoS Pathog. 2016;12: e1006093. doi:10.1371/journal.ppat.1006093

3. Balla KM, Andersen EC, Kruglyak L, Troemel ER. A Wild C. Elegans Strain Has Enhanced Epithelial Immunity to a Natural Microsporidian Parasite. PLoS Pathogens. 2015;11: e1004583. doi:10.1371/journal.ppat.1004583

4. Reinke AW, Balla KM, Bennett EJ, Troemel ER. Identification of microsporidia host-exposed proteins reveals a repertoire of rapidly evolving proteins. Nat Commun. 2017;8: 14023. doi:10.1038/ncomms14023

5. Wadi L, El Jarkass HT, Tran TD, Islah N, Luallen RJ, Reinke AW. Genomic and phenotypic evolution of nematode-infecting microsporidia. bioRxiv. 2022; 2022.08.28.505597. doi:10.1101/2022.08.28.505597
