# Supplementary material for: High-throughput phenotyping of infection by diverse microsporidia species reveals a wild C. elegans strain with opposing resistance and susceptibility traits
Source: PLoS Pathog. 2023 Mar 9;19(3):e1011225. doi: 10.1371/journal.ppat.1011225 (PMC10030041; doi:10.1371/journal.ppat.1011225)
Supplement: S2 Table — (DOCX) [file ppat.1011225.s022.docx]

**Table S2. PhenoMIP microsporidia infection doses.**

| **Microsporidia species** | **Microsporidia**  **strain** | **Infection**  **round** | **Spores per cm^2^ media** | | |
| --- | --- | --- | --- | --- | --- |
|  |  |  | **Low** | **Medium** | **High** |
| *Nematocida parisii* | ERTm1 | 1 | 8,000 | 16,000 | 32,000 |
|  |  | 2-4 | 13,269 | 26,539 | 53,078 |
|  |  | **Follow-up infections** |  | 26,539 | 53,078 |
| *Nematocida ausubeli* | ERTm2 | 1 | 26,539 | NA | 53,078 |
|  |  | 2-4 | 26,539 | 39,808 | 53,078 |
|  |  | **Follow-up infections** |  | 17,692 | 53,078 |
| *Nematocida ironsii* | ERTm5 | 1 | 22,116 | 44,232 | 88,464 |
|  |  | 2-4 | 30,692 | 61,924 | 123,849 |
|  |  | **Follow-up infections** |  | 61,924 | 123,849 |
| *Nematocida ferruginous* | LUAm1 | 1 | 88,464 | 176,928 | 353,857 |
|  |  | 2-3 | 159,235 | 318,471 | 636,942 |
|  |  | 4 | 159,235 | 318,471 | 751,592 |
|  |  | **Follow-up infections** |  | 318,471 | 751,592 |
|  | LUAm3 | **Follow-up infections** |  | 353,857 |  |
| *Nematocida cider* | AWRm77 | **Follow-up infections** |  | 141,542 |  |

* effective dose of spores used in retests and additional infections
