# Supplementary material for: High-throughput phenotyping of infection by diverse microsporidia species reveals a wild C. elegans strain with opposing resistance and susceptibility traits
Source: PLoS Pathog. 2023 Mar 9;19(3):e1011225. doi: 10.1371/journal.ppat.1011225 (PMC10030041; doi:10.1371/journal.ppat.1011225)
Supplement: S3 Table — (DOCX) [file ppat.1011225.s023.docx]

**Table S3. Wild isolate mapping microsporidia infection doses.**

| **Wild isolate strain(s)** | **Microsporidia strain** | **Infection time (hours)** | **Infection round** | **Spores per cm^2^ media** |
| --- | --- | --- | --- | --- |
| JU1400, MY1 | *Nematocida ferruginous* | 72 | 1-2 | 350,318 |
|  |  |  | 3 | 445,859 |
| JU1400 | *Nematocida ironsii* | 72 | 1 | 178,343 |
|  |  |  | 2-3 | 254,777 |
| JU1400 | *Nematocida ironsii* | 96 | 1-2 | 509,554 |
| CB4856 | *Nematocida ironsii* | 72 | 1 | 178,343 |
|  |  |  | 2-3 | 254,777 |
