# Supplementary material for: High-throughput phenotyping of infection by diverse microsporidia species reveals a wild C. elegans strain with opposing resistance and susceptibility traits
Source: PLoS Pathog. 2023 Mar 9;19(3):e1011225. doi: 10.1371/journal.ppat.1011225 (PMC10030041; doi:10.1371/journal.ppat.1011225)
Supplement: S4 Table — (DOCX) [file ppat.1011225.s024.docx]

**Table S4. NIL strain chromosome I region composition.**

| **Strain** | **I:491,763** | **I:1,051,810** | **I:1,567,450** | **I:2,012,869** | **GFP Status**  **I-2,851,135** |
| --- | --- | --- | --- | --- | --- |
| AWR134 | JU1400 | JU1400 | JU1400 | -- | + |
| AWR135 | JU1400 | JU1400 | JU1400 | JU1400 | - |
| AWR136 | JU1400 | JU1400 | JU1400 | JU1400 | + |
| AWR137 | JU1400 | JU1400 | JU1400 | JU1400 | - |
| AWR138 | JU1400 | -- | -- | -- | + |
| AWR139 | JU1400 | JU1400 | JU1400 | JU1400 | - |
| AWR140 | JU1400 | JU1400 | -- | -- | + |
| AWR141 | JU1400 | JU1400 | JU1400 | JU1400 | - |
| AWR142 | JU1400 | JU1400 | -- | -- | + |
| AWR143 | JU1400 | JU1400 | JU1400 | JU1400 | - |
