# Supplementary material for: High-throughput phenotyping of infection by diverse microsporidia species reveals a wild C. elegans strain with opposing resistance and susceptibility traits
Source: PLoS Pathog. 2023 Mar 9;19(3):e1011225. doi: 10.1371/journal.ppat.1011225 (PMC10030041; doi:10.1371/journal.ppat.1011225)
Supplement: S9 Table — (DOCX) [file ppat.1011225.s029.docx]

**Table S9. Gene classes and domains used for enrichment analyses.**

| Domain/ Gene Family | Databse | Wormbase gene class OR Pfam ID |
| --- | --- | --- |
| F-box | Wormbase | fbxa, fbxb, fbxc |
| MATH (meprin or Traf homology) or BATH (BTB and MATH domain-containing) | Wormbase | math, bath |
| PALS (protein containing ALS2CR12 signature) | Wormbase | pals |
| C-type lectins | Wormbase | clec |
| DUF713 | Pfam | PF015218 |
| DUF684 | Pfam | PF05075 |
| chil | Wormbase | chil |
| Histone | Pfam | PF00125 |
| Nematode cuticle collagen N-terminal domain | Pfam | PF01484 |
| skr (Skp1-related) | Wormbase | Skr |
| Other (Any genes not in the domain or gene family above) | - | - |
