# Supplementary material for: High-throughput phenotyping of infection by diverse microsporidia species reveals a wild C. elegans strain with opposing resistance and susceptibility traits
Source: PLoS Pathog. 2023 Mar 9;19(3):e1011225. doi: 10.1371/journal.ppat.1011225 (PMC10030041; doi:10.1371/journal.ppat.1011225)
Supplement: S11 Table — (DOCX) [file ppat.1011225.s031.docx]

**Table S11. Published data sets from WormExp used in this study.**

| Chemical/Pathogen | WormExp Term |
| --- | --- |
| Cadmium | - down by Cadmium (Huffman) - UP by Cadmium (Huffman) |
| Dichlorvos | - Down by high dichlorvos - UP by high dichlorvos |
| Fenamiphos | - Down by fenamiphos high - UP by fenamiphos high |
| Mercury | - Down by mercury high 8H - UP by mercury high 8H |
| Ivermectin (IVM) | - Down by 1ng/ml ivermectin - UP by 1ng/ml ivermectin |
| Mefloquine | - Down by mefloquine high - UP by mefloquine high |
| Heat Shock F3 | - Down HS F3 vs. CTL F3 - UP HS F3 vs. CTL F3 |
| Acrylamide | - Down by high acrylamide 8H - UP by high acrylamide 8H |
| Multi-walled carbon nanotubes (MWCNT) | - Down by MWCNT 24H vs. 0H - UP by MWCNT 24H vs. 0H |
| Tert-butyl hydrogen peroxide | - down by tert-butyl hydrogen peroxide (Oliveira) - UP by tert-butyl hydrogen peroxide (Oliveira) |
| *Bacillus thuringiensis* strain BT247 | - down by B. thuringiensis at 6h (BT247, 1:2) (Yang) - down by B. thuringiensis at 6h (BT247, 1:2) (Yang) |
| *Bacillus licheniformis* strain 141 | - down by B. licheniformis 141 - UP by B. licheniformis 141 |
| *Drechmeria coniospora* | - Down D. coniospora 12h, RNASeq - UP D. coniospora 12h, RNASeq |
| *Pseudomonas aeruginosa* strain PA14 | - down by PA14, 24h - UP by PA14, 24h |
| Cry5B toxin | - down by Bt toxin,Cry5B - UP by Bt toxin,Cry5B |
| *Candida albicans* | - down by C. albicans (Pukkila-Worley) - UP by C. albicans (Pukkila-Worley) |
| *Lactobacillus rhamnosus* strain CNCM I-3690 | - Down fed with L. rhamnosus CNCM I-3690 vs. OP50,3 days - UP fed with L. rhamnosus CNCM I-3690 vs. OP50,3 days |
| *Staphylococcus aureus* | - down by S. aureus (Bond) - UP by S. aureus (Bond) |
| *Serratia marcescens* | - Down infected by S. Marcescens - UP on S. marcescens |
| *Xenorhabdus nematophila* | - Down infected by X. Nematophila - UP on X. nematophila |
