# Supplementary material for: High-throughput phenotyping of infection by diverse microsporidia species reveals a wild C. elegans strain with opposing resistance and susceptibility traits
Source: PLoS Pathog. 2023 Mar 9;19(3):e1011225. doi: 10.1371/journal.ppat.1011225 (PMC10030041; doi:10.1371/journal.ppat.1011225)
Supplement: Attachment — Submitted filename: Response letter_plos path_final.pdf [file ppat.1011225.s036.pdf]

We thank the reviewers for their comments. We have provided a revised manuscript (where all changes are highlighted) to address the concerns raised by the reviewers. These changes have been incorporated into the manuscript (including additional supplemental figures and tables) and we have provided a point-by-point response to each of the reviewer's comments.

REVIEWER COMMENTS

Part I - Summary
Please use this section to discuss strengths/weaknesses of study, novelty/significance, general execution and scholarship.

Reviewer #1: In this manuscript, Mok et al perform a large-scale survey of how c. Elegans fitness is affected by diverse microsporidia pathogens. The large-scale survey identified multiple leads, of which the authors further characterized two elegans-microsporidia interactions. Overall, the main conclusions are well supported by the presented data, though there are several places where additional clarification would be helpful to readers.

Reviewer #2: The manuscript by Mok et al., describes high-throughput phenotyping of C. elegans wild isolates and their interactions with various species of microsporidia from the genus Nematocida using multiplexed competition assays. While several potential new interactions were identified, the most intriguing finding is the opposing phenotype of the isolate JU1400, which is sensitive to epidermis-infecting N. ferruginous, but resistant to intestine-infecting *N. ironsii*. Genetic mapping indicates that these phenotypes are likely to be driven by separate alleles. Additionally, the authors present a transcriptional analysis showing that transcriptional response to microsporidia species is largely conserved and similar to IPR, with minor strain-specific differences, but no significant correlation with the resistant or susceptibility phenotypes. Overall, it's an ambitious attempt to understand how pathogens can shape host evolution, and this approach may be used in the future for the high throughput study of other C. elegans isolates interactions with their natural pathogens. The presentation of the data and the conclusions drawn can be improved as described with more specific comments below.

Reviewer #3: In this manuscript by Mok et al, the authors report a high-throughput phenotyping of infection by diverse microsporidia species among wild isolates and identified one strain, JU1400 with two opposing resistance and susceptibility traits. They further conducted genetic mapping and revealed multiple loci responsible for the interactions JU1400 with microsporidia. Lastly, they conducted RNAseq to examine the transcriptomes of JU1400 in response to microsporidia infection.

Part II – Major Issues: Key Experiments Required for Acceptance

Reviewer #1: None to note

Reviewer #2: 1. An obvious limitation is that the authors narrow down the genetic interval for one interaction to 41 protein coding changes (is there a list included in the supplement?), but present no attempt to identify the causative genes. Did the authors try to test by RNAi the best candidates in the interval on chromosome I and see whether they modulate susceptibility of JU1400 towards N. ferruginous. Alternatively, could the authors put together a hypothetical

model based on known/predicted functions of these genes.

We have now added Supplementary Table S5 which contains the proteins and coding variants that are present in both JU1400 and MY1 compared to N2. We have modified the results section to reference this table. We strongly feel that identifying the causative gene in JU1400 is outside the scope of this paper, although we plan on performing studies to identify this gene in the future.

2. It has been shown that activation of IPR in the hypodermis is sufficient to provide protection from intestine infecting N. parisii (https://journals.plos.org/plosgenetics/article?id=10.1371/journal.pgen.1010314). Considering that JU1400 displays different sensitivity towards intestine and hypodermis infecting microsporidia, what happens if JU1400 is exposed simultaneously or sequentially to both *N. ironsii* and N. ferruginous? This would be a nice addition to the co-infection experiments performed in this study.

Although this suggested experiment could be performed, we feel that the additional data generated wouldn't add to our overall conclusions. We have already performed coinfection studies in JU1400 infected with *N. ausubeli* and *N. ironsii*, showing that infection with *N. ironsii* does not lead to increased clearance of *N. ausubeli* (Figure 5 and Supplementary Figure S11). Unlike *N. ironsii*, *N. ferruginous* does not proliferate in the intestine (Wadi et al, *bioRxiv*, 2022). Thus, we think it unlikely that the coinfection of *N. ironsii* would lead to less *N. ferruginous*, at least in a JU1400 specific manner. Studies with other pairs of microsporidia infecting a variety of hosts have been previously performed, with infection of one species sometimes preventing growth of the other, though this is not always the case (Willis et al, *Exp Suppl*, 2022).

3. In Fig.3 E,F a single early timepoint is presented, but later time points are also required to further support the proposed susceptibility to N. ferruginous infection due to a weakened tolerance (in light of other results presented in the paper too).

In Figure 3E, we provide data showing the number of *N. ferruginous* sporoplasms after 3 hours of infection. In Figure 3F, we quantify *N. ferruginous* parasite burden at 72 hours. Thus we have examined both an early and a late timepoint. We have now modified the figure legend for Figures 3E and F to make it clearer which timepoints we examined.

Reviewer #3: This manuscript is very data-heavy. The authors have spent huge amount of efforts in getting all these data. However, the presentation of the results is often not very clear. I suggest that the authors need to revise their figures so that they can be easily followed and understood by the readers. For examples, in the text, the authors said that they identified 13 strains with phenotypes (line 129). However, it is not clear in Fig. 2, which are the 13 strains?

We have now provided a revised version of Figure 2 which we hope improves the clarity. This new version of Figure 2 shows the 16 *C. elegans* strain-microsporidia interactions we identified as having relative increase or decrease of host fitness upon microsporidia infection. We have also moved the previous version of Figure 2 to be Supplementary Figure S2.

In Fig. 3A and B, it is very difficult to distinguish all the different lines. In all the Bar charts, there are too many lines in the background. I suggest removing them and make the charts cleaner.

We have now removed the horizontal lines from our main figures to make the charts easier to understand.

For the susceptibility mapping, the authors mapped to ~700 Kb region on chromosome 1. The authors only described the number of the genes in this region but did not tell us what genes they are? (line 258-259). It will be nice to at least discuss the potential genes within this locus that might be crucial for the susceptibility.

As mentioned in our response to reviewer #2, We have now included a Supplementary Table S5 which contains the proteins and coding variants that are present in both JU1400 and MY1 compared to N2. As there are still many potential candidates, we feel that discussing potential genes within this region would be too speculative.

For the RNAseq section, the authors mainly focused on the number of the DEGs in each of the conditions, which is not very informative. I suggest that the authors providing a summary of GO term enrichment analyses for the different samples and draw some conclusions. I also feel that the authors should identify a couple interesting targets from their RNAseq data and follow up with functional tests. Currently the RNAseq section is lack of focus. Readers do not get much information out of these huge datasets.

We have now added Supplementary Tables S13 and S14 which includes GO term and KEGG pathway enrichment for our RNA-seq datasets. We have updated the results and methods section to include these data. We strongly feel that functionally characterizing candidates identified from our RNA-seq data is outside the scope of this paper.

Part III – Minor Issues: Editorial and Data Presentation Modifications
Please use this section for editorial suggestions as well as relatively minor modifications of existing data that would enhance clarity.

Reviewer #1: Initial experiment to assess wild isolate responses to diverse microsporidia:

The authors looked at the fitness of 22 wild-isolates and the lab strain VC20019 after exposure to four microsporidia species at four different spore concentrations. Pooled L1s were exposed to spores for 72h and bleach synchronized, allowed one generation to recover, and infected again. This two generation cycle was repeated four times for all conditions except N. ausubeli medium dose. The authors maintained worms in starvation-free conditions for two weeks prior to starting the infection experiments. Prior to infection experiments, worms were bleach synchronized and arrested in a starvation state, which the worms went through during each generation of the infection experiment. Does larval arrest after bleach synchronization have an effect on microsporidia infection? Does this vary across wild isolates? I'm mostly curious because the authors paid close attention to preventing starvation for two weeks prior to starting this assay only to expose them to starvation for the immediate generation prior to the infection experiment and all subsequent generations. I understand that this might be the only way to properly initiate

infection experiments, but am curious as to the author's rationale for preventing starvation prior to inducing starvation conditions.

The choice to induce a short-term starvation of the L1 animals is to allow for synchronization of the animals so that the microsporidia infection is initiated in the same developmental stage of animals. We have previously shown that infection of embryos hatched on a plate with *E. coli* get infected to similar extent as populations of animals that were arrested as L1s and then infected (Tamim El Jarkass et al, *eLife*, 2022). This approach of using starved L1s has also been previously used with microsporidia infection of *C. elegans* wild isolates (Balla et al, *PLOS pathogens*, 2015). Although we can not rule out that in some wild isolates this short starvation may have an impact on microsporidia infection, our protocol only induces a starvation of 8-14 hours and an article on natural variation to starvation resistance in *C. elegans* (Webster et al, *eLife*, 2022), reported minimal changes in wild isolate survival following 24 hours of L1 starvation. We have now modified the results in lines 104-110 to clarify these points.

The authors identified several strain specific interactions from their large initial experiment to further characterize. It should be noted, and i mention this below, that of the two interactions the authors followed up, one interaction was found to be the opposite of the original observation. Though this does not affect the conclusions of the manuscript, I think there should be more discussion about this point. Despite the author's claim on lines 390-391, I was not convinced that PhenoMIP is high-throughput approach to identify variants associated with pathogen susceptibility.

Our initial observation from our PhenoMIP experiment is that JU1400 has severely reduced fitness when infected with *N. ferruginous* and a moderate reduction of fitness when infected with *N. ironsii* (Figure 3A). In our follow up experiments using embryo counts as a proxy for fitness, we observed that at 72 hours there was no difference in reduction of embryos during infection with *N. ironsii* (Figure 4A) and significantly less reduction of embryos in JU1400 at 96 hours (Figure 4B). However, we also observed a decrease in embryos when JU1400 was infected with *N. ironsii* compared to N2 or VC20019 (Figure S4B). We also performed MIP-MAP experiments using similar conditions to what was done in our initial PhenoMIP experiment. These experiments showed that JU1400 regions were selected for at both 72 hours (figure S13B) and at 96 hours (Figure 6B). Regions on both chromosome II and chromosome V were selected for at both times, but only the region on the X chromosome was selected for at 96 hours. In addition to our experiments on host fitness, we also provide strong evidence that there is resistance to *N. ironsii* in JU1400 due to its ability to clear the parasite. Thus, our evidence is supportive of a model where JU1400 is resistant to *N. ironsii* infection and has increased fitness compared to control strains at a 96-hour time point. Our data is inconclusive about the impact of *N. ironsii* on JU1400 at 72 hours as we have conflicting results depending on the experiment.

We have now modified the discussion in lines 433-449 to make it clearer what we can conclude from our data from these experiments. We have also modified how we refer to the phenotypes detected by the PhenoMIP assay, referring to them as having a relative increase or decrease in host fitness.

We disagree with the reviewer's assertion that PhenoMIP is not able to identify variants associated with pathogen susceptibility. We confirmed that 75-87.5% (depending on the Z score cut off) of the strains that we tested individually have the phenotype identified in our PhenoMIP assays. However, our results with JU1400 and *N. ironsii* infection suggest that 96

hours may be a more appropriate time point in which to identify resistant strains. This limitation of our current assay and possible improvement has been added to the discussion in lines 449-452.

N. ferruginous section for ju1400:
The authors performed a 3hr infection pulse to determine if there were measurable differences between N2 and Ju1400, there were none, so they concluded JU1400 had a tolerance issue. Tolerance seems like a broad and unspecific term that the authors tried to explore with transcriptional profiling, which, as discussed below, did not add much clarity to the author's experimental observations.

We have now better defined how tolerance has been discussed in the literature in lines 391-392 of the discussion. We disagree with the reviewer that transcriptional profiling does not add to our understanding of the lack of tolerance in JU1400. We observe a transcriptional profile that is specific to JU1400 infected with *N. ferruginous* which is similar to worms grown on chemical toxins. Although these transcriptional experiments do not reveal the mechanism by which JU1400 lacks tolerance, they do provide insight on the consequences of this lack of tolerance.

The authors used MIP-MAP to determine the genomic region associated with the increased sensitivity of JU1400 to N. Ferruginous and *N. ironsii*. The different pathogens are associated with distinct genomic loci and the authors validate the major N. Ferruginous susceptibility locus on chromosome I by constructing NILs. I am curious if any of the differentially expressed genes in response to this pathogen map to this region of the genome and might be able to inform the causative allele? This is something that I thought was missing from the transcriptional analysis.

We have now analyzed the genes that have coding variants that are present in both JU1400 and MY1 compared to N2. None of these genes are differentially regulated in our transcriptional data sets. We have now added this result to lines 377-380

Later in the manuscript the authors describe transcriptional profiling of infected JU1400 exposed to N. ferruginous for 48h. Lines 308-310 the authors point to a striking observation of differential expressed genes with collagen domains without elaborating on why it was striking. I imagine one reason might be that N. ferruginous infects muscle and epidermal cells? Could another reason be that infected animals are at a different developmental stage and express a different complement of collagen-containing proteins? Overall the presentation of the transcriptional profiling experiments was quite confusing and did little to support the claim that JU1400 is less tolerant to N. ferruginous infection than N2/VC20019/AWR133, which was made evident by the infection fitness assay.

We agree with the reviewer that the change in developmental timing is a possible cause of differential regulation of collagen-containing proteins. We have now added to the discussion in lines 423-426 that the lack of tolerance in JU1400 infected with *N. ferruginous* could alter developmental timing.

N. ironsii and JU1400:
In the initial experiment, Ju1400 was identified to be more susceptible to *N. ironsii*. Upon further investigation, the opposite turned out to be true. I think it would help readers to underscore this phenotypic switching in the discussion instead of glossing over it on lines 345-346. From the data presented, it is unclear why JU1400 would appear to be sensitive to *N. ironsii* in their large assay, given it has the same number of embryos as N2 72hpi, while VC20019 does not show the same trend in the assay (figure 2).

This comment has been addressed in our response above.

It seems that the better control for Figure 4A-B would have been VC20019 and not N2, with the unsaid understanding that N2 and VC20019 should behave similarly, despite them being different strains.

In Supplementary Figure S5 we tested VC20019 and N2 against *N. ironsii* and we did not observe any difference, suggesting that these two strains are impacted to a similar extent by *N. ironsii.* We have provided additional information on the choice of VC20019 vs. N2 in response to reviewer #2 below.

As noted above the author's due diligence following up the JU1400 *N. ironsii* interaction revealed that JU1400 is resistant to this pathogen, as measured by lower pathogen load after a brief infection and embryo counts at 96hpi. The specificity that Ju1400 has for clearing *N. ironsii* is an interesting result that lays the foundation for future studies to explore the molecular details of this interaction.

We agree with the reviewer, and we plan on investigating the molecular basis behind this resistance in future experiments.

While I appreciate the authors attempted transcriptional profiling to determine why JU1400 was more tolerant to N. ferruginous, as presented, it was unclear what these experiments added to this manuscript. Nevertheless, these data will likely help inform future investigations into these host-pathogen responses.

We discuss above how the transcriptional profiling demonstrates the consequences of lack of tolerance in JU1400 animals infected with *N. ferruginous*. We agree that this transcriptional data will also be useful for further studies.

Figure 2: I appreciate the concise summary of a lot of experimental data. I do think this figure can be improved by also showing the strain trajectories for the two microsporidia species that the authors follow up in the rest of the manuscript. Perhaps highlighting the focal C. Elegans strains JU1400 in each condition, as it is the exemplary strain showing multiple species-specific interactions.

As mentioned above in our response to reviewer #3, we have now modified Figure 2 to make the experimental results of our PhenoMIP experiments more concise.

Figure 3B seems to suggest that MY1 is more fit in the presence of high doses of N. ausubeli (solid thick orange lines) than in mock infection conditions. Do the authors have any idea why this might be the case or am I interpreting this figure incorrectly?

Although these lines are slightly higher than the mock infected conditions, MY1 does not meet our statistical threshold for causing a phenotype. This was previously shown in Figure 2 which we have now moved to Supplementary Figure 2.

Figure 3D, 4A-B, 6D, : it is unclear exactly how the authors arrived at embryo counts per worm and to what these counts were normalized. I am assuming these counts correspond to embryos within gravid animals, but I am just guessing.

Our approach to determining embryo counts was to count the number of embryos per animal in each condition. We then normalized these counts to the corresponding uninfected strain. As some of the wild strains, including JU1400, had different embryo counts compared to N2, this normalization was necessary to capture the impact of infection on host fitness. We have now updated the methods with an "Embryo analysis and normalization" section (lines 605-614) to better describe how the embryo counts were performed and normalized.

Figure 3 A/B could look a bit cleaner by just including the microsporidia strains of interest

We have now provided a modified version of Figure 3 A and B to improve the clarity of this data.

Reviewer #2:

1. In terms of the interactions observed for microsporidia with various wild isolates, can the authors comment whether resistant and susceptible nematode strains genetically cluster together? Also, are there any geographical region parameters (like temperature, humidity, elevation etc) that might shape the interaction observed between the microsporidia species and C. elegans wild isolates?

   Although this is a very interesting question, we have limited data for which to make these conclusions. We do think that these questions would be interesting to look at with a larger set of strains, similar to what has been done comparing wild isolates to different environmental conditions (Evans et al, G3, 2017).

2. In figure 3A-B and figure S2, why two lines are represented for each condition? Are these two different replicates? I would recommend that the authors show averaged or most representative line graph in this case, as it is difficult to read this graph due to crowding of multiple lines.

As mentioned above in our response to reviewer #1, we have now modified Figures 3A and B to improve their clarity.

3. How can the authors account for the fitness disadvantage of JU1400 in the competition assay upon *N. ironsii* infection? Can the authors rule out technical limitations such as variability in growth and brood size in the conditions used for the experiment rather than short-term/long-term effects of infection? Accordingly, table 1 can be a bit misleading, for example, it mentions JU1400 as susceptible to both *N. ironsii* and N. ferruginous, so it might be good to clarify this on this table (ie add an asterisk/footnote or something).

We have responded above to reviewer #1 about the differences we see in our different assays when JU1400 is infected with *N. ironsii* and as we mentioned it is not clear why we see conflicting results ranging from a decrease to increase in fitness. What is clear that there is a relative increase of fitness when JU1400 is infected with *N. ironsii* at 96 hours, so this variability that we see is more prominent at this earlier time point. We have now modified Table 1 to make it clear by labeling this as a change in host fitness.

4. The presentation of the transcriptional analysis of JU1400 exposed to various nematocidal species can be improved. I felt that clarity is lacking on what are the take home messages in the context of the main question ie why JU1400 shows opposing phenotypes. The comparison to drugs is highly speculative and can be moved to the supplement.

As we mentioned in our response to reviewer #1, we feel that this transcriptional analysis does provide information on the opposing phenotypes. In response to *N. ironsii* we don't observe an elevated immune response, and when JU1400 is infected with *N. ferruginous*, we observe the consequences of lacking tolerance. We believe that the comparison of this transcriptional profile to different chemical toxins is useful and we have now modified the discussion in lines 419-421 so that our description of this result is not interpreted as being highly speculative.

Additionally, this analysis might fit more before the section on genetic mapping, as it seems to give to the paper an abrupt ending otherwise and does not link to the mapping at all. The scale and colours in Figure 7C and 7D does not allow for clear visualisation of genes that are mentioned in the key.

We have now ended the results section by linking the transcriptional data back to the mapping. We have now added Supplementary Table S10 which allows for clear visualization of the domain representation in each of our groups.

5. The choice of VC20019 (from the mullion mutation project) as "reference" warrants justification. This is a mutagenized strain so do the authors know that it has similar development time and brood size to N2 and JU1400 ?

The choice of the VC20019 strain was previously described and characterized in Mok et al. Genetics, 2017, and Mok et al, G3, 2020. This strain develops similar to N2 and also has a similar fecundity. This strain also has 269 variants compared to N2 that allow it to be easily distinguished using molecular inversion probes. As described above we directly compared N2 and VC20019 during infection with different microsporidia species in Figure S3-S5. We did not

observe a difference in either the host fitness or amount of pathogen in any of these experiments. We have now added a sentence to the results in line 107-109 to the results better justifying the choice of this strain.

Other Comments:
Line 53 remove extra spacing
Line 680 correct FISHs to FISH
Line 714 italicize N
Line 983 italicize pathogen name
Some inconsistency throughout in the use of spaces and hyphenating words, especially in materials and methods section. Please check.

We have made the requested changes and have thoroughly reviewed the manuscript to identify other typos.

Reviewer #3: (No Response)
